# Benzoquinoline Derivatives: A Straightforward and Efficient Route to Antibacterial and Antifungal Agents

**DOI:** 10.3390/ph14040335

**Published:** 2021-04-06

**Authors:** Vasilichia Antoci, Liliana Oniciuc, Dorina Amariucai-Mantu, Costel Moldoveanu, Violeta Mangalagiu, Andreea Madalina Amarandei, Claudiu N. Lungu, Simona Dunca, Ionel I. Mangalagiu, Gheorghita Zbancioc

**Affiliations:** 1 Faculty of Chemistry, Alexandru Ioan Cuza University of Iasi, 11 Carol 1st Bvd, 700506 Iasi, Romania; vasilichia.antoci@uaic.ro (V.A.); lili_oniciuc@yahoo.com (L.O.); dorina.mantu@uaic.ro (D.A.-M.); costel.moldoveanu@uaic.ro (C.M.); ionelm@uaic.ro (I.I.M.); 2 Institute of Interdisciplinary Research—CERNESIM Centre, Alexandru Ioan Cuza University of Iasi, 11 Carol I, 700506 Iasi, Romania; violeta.mangalagiu@uaic.ro; 3 Faculty of Biology, Alexandru Ioan Cuza University of Iasi, 11 Carol 1st Bvd, 700506 Iasi, Romania; aamarandei16@yahoo.com; 4 Department of Surgery, Emergency Clinical Hospital, 810325 Braila, Romania

**Keywords:** antibacterial, antifungal, molecular docking, mechanism of action, ADMET, SAR/QSAR, benzo[f]quinoline, salts, cycloadducts

## Abstract

We report here the design, synthesis, experimental and in silico evaluation of the antibacterial and antifungal activity of some new benzo[f]quinoline derivatives. Two classes of benzo[f]quinolinium derivatives—(benzo[f]quinolinium salts (**BQS**) and pyrrolobenzo[f]quinolinium cycloadducts (**PBQC**)—were designed and obtained in two steps via a direct and facile procedure: quaternization followed by a cycloaddition reaction. The synthesized compounds were characterized by elemental and spectral analysis (FT-IR, ^1^H-NMR, ^13^C-NMR). The antimicrobial assay reveals that the **BQS** salts have an excellent quasi-nonselective antifungal activity against the fungus *Candida albicans* (some of them higher that the control drug nystatin) and very good antibacterial activity against the Gram positive bacterium *Staphylococcus aureus*. The **PBQC** compounds are inactive. Analysis of the biological data reveals interesting SAR correlations in the benzo[f]quinolinium series of compounds. The in silico studies furnished important data concerning the pharmacodynamics, pharmacokinetics and ADMET parameters of the **BQS** salts. Studies of the interaction of each **BQS** salt **3a–o** with ATP synthase in the formed complex, reveal that salts **3j**, **3i,** and **3n** have the best fit in a complex with ATP synthase. Study of the interaction of each **BQS** salt **3a-o** with TOPO II in the formed complex reveals that salts **3j** and **3n** have the best-fit in complex with TOPO II. The in silico ADMET studies reveal that the **BQS** salts have excellent drug-like properties, including a low toxicity profile. Overall, the experimental and in silico studies indicate that compounds **3e** and **3f** (from the aliphatic series), respectively, and **3i**, **3j** and **3n** (from the aromatic series), are promising leading drug candidates.

## 1. Introduction

According to the WHO, infectious diseases, especially those caused by bacterial (Gram positive and Gram negative) and fungal microorganisms, have become a serious threat to the global health system, being responsible for 22% of all deaths and 27% of disability-adjusted life years worldwide [1]. Antibiotics play a key central role in antimicrobial therapy, and are one of the most effective and successful weapons against different microorganisms. However, the overuse and misuse of antibiotics have led to widespread drug resistance (DR), multi-drug resistance (MDR) and extensive-drug-resistance (EDR), creating an urgent need for new antimicrobial agents [1,2,3,4].

**Scheme 1:**
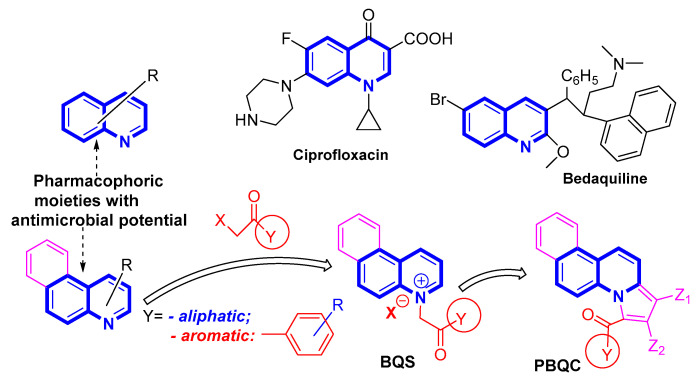
Design in the class of benzoquinolines derivatives with antimicrobial activity.

Quinoline and its benzo-fused derivative, benzoquinoline, are crucial scaffolds in medicinal chemistry and they have been reported to possess a large variety of biological activities which include antiplasmodial and antimalarial, antitubercular, antibacterial, antifungal, anti-HIV, anticancer, antinociceptive and anti-inflammatory, antipsychotic, analgesic, anti-Alzheimer’s, antihypertensive properties, etc. [5,6,7,8,9,10,11,12,13,14,15,16,17]. Moreover, the quinoline pharmacophore has proved to be one of the most effective motifs used in antibacterial and antifungal therapy, and many of the existing drugs on the market incorporate a quinoline scaffold e.g., ciprofloxacin (antibacterial), bedaquiline (antitubercular), etc. [18,19], (Scheme 1).

Taking into consideration the above considerations and our expertise in the field of antibacterial and antifungal agents [13,15,20,21,22,23,24,25], we report here the design, synthesis, molecular docking, antibacterial and antifungal evaluation of some new benzo[f]quinoline derivatives.

## 2. Results and Discussion

### 2.1. Desingn and Chemistry

Taking into consideration the abovementioned data, we decided to combine the pharmacophoric antimicrobial capabilities (Scheme 1) of quinoline and its benzo derivatives [26] and also to convert them in salts, in view of the fact that salts usually have better antimicrobial activity and also better pharmacokinetic properties; we also had in mind increasing the number of fused cycles from three in benzo[f]quinoline to four and to see their influence in regards to the antimicrobial activity. In equal measure we were interested in seeing the influence on antimicrobial activity of the substituents on the quaternized nitrogen, taking into account two series an aliphatic and aromatic one. As a result, two series of benzo[f]quinoline derivatives was designed and synthesized: **BQS** salts and **PBQC** cycloadducts (Scheme 1). 

**Scheme 2:**
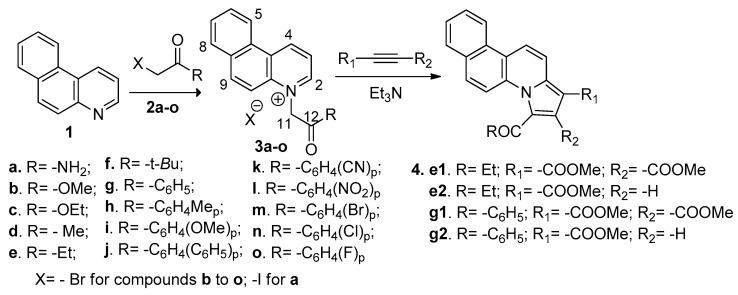
Reaction pathways to obtain benzo[f]quinoline salts **3a–o** and cycloadducts **4e** and **4g**.

In order to synthesize our fused quinoline derivatives we used a direct and facile two step procedure: quaternization of nitrogen heterocycles followed by a cycloaddition reaction. Using an adaptation of the setup procedure from the literature [27,28,29], in the first step, we performed a quaternization reaction of benzo[f]quinoline (**1**) with variously activated α-halocarbonyl compounds **2a**–**o** (such as: 1-bromo-alkyl-2-one, 2-iodoacetamide, (un)substituted phenacyl bromides), when the corresponding benzo[f]quinolinium quaternary salts **BQS 3a**–**o** were obtained (Scheme 2). The next step consists in a Hüisgen [3 + 2] dipolar cycloaddition of the benzo[f]quinolinium ylides (generated in situ from the corresponding benzo[f]quinolinium quaternary salts **BQS 3a**–**o**, in alkaline medium) to alkyne dipolarophiles (non-symmetrically or symmetrically substituted *Z*-alkynes, methyl propiolate and dimethyl acetylenedicarboxylate, DMAD), when the corresponding fused pyrrolobenzo[f]quinolinium cycloadducts **PBQC 4**, were obtained (Scheme 2). Initially, the cycloaddition reactions were performed in the case of salts **3e** (aliphatic) and **3g** (aromatic), and the obtained cycloadducts **PBQC** (**4e1**, **4e2**, **4g1**, and **4g2**) was subject to antimicrobial assay. Because the antimicrobial activity of the obtained cycloadducts **PBQC 4e** and **4g**, was negligible, we did not continue our studies in this direction.

The structures of compounds were proved by elemental and spectral analysis (FT-IR, ^1^H-NMR, ^13^C-NMR, and two-dimensional experiments 2D-COSY, HMQC, HMBC). The main data furnished by FT-IR and NMR spectral analysis are listed in Table 1. In the FT-IR spectra of **BQS** salts **3a**–**o**, the most important signals are those one of carbonyl groups. In the aromatic **BQS** salts **3g**–**o**, the signals corresponding to carbonyl ketone group are situated between 1717 cm^−1^ (R = NO_2_, **3l**) and 1673 cm^−1^ (R = OCH_3_, **3i**), in accordance with the electronic effects of substituents on the 4-position of a phenyl ring (electron-withdrawing for NO_2_ and electron-donating for OCH_3_). In the aliphatic series (**BQS** salts **3a–f**), the signals corresponding to carbonyl group are situated between 1626 cm^−1^ (amide CO, **3a**) and 1743 cm^−1^ (carboxymethyl CO, **3b**), in accordance with the structures of the compounds (amide and aliphatic ester). In the ^1^H-NMR spectra the most important signals are those of the H-2, H-4, and H-11 (methylene (–CH_2_, –) hydrogen) atoms. As it can be seen in Table 1 the H-11 methylene hydrogen atoms appear at an unusual very high chemical shift, around 6.50 ppm in the aliphatic series and around 7.00 ppm in the aromatic series. This is in accordance with the powerful electron-withdrawing effect exerted by the positive α–endocyclic nitrogen atom of the benzo[f]quinoline ring. The aromatic/aliphatic difference of about 0.50 ppm is because of the supplementary electron-withdrawing effect exerted by the aromatic phenyl ring. The H-2 and H-4 protons from the pyridine ring appear at high chemical shift, around 10.25 ppm H-4 (positive γ–endocyclic nitrogen) and around 9.50 ppm H-2 (positive α–endocyclic nitrogen).

The ^13^C-NMR spectra of **BQS** salts **3a**–**o** also confirm the structurse and are in accordance with the FT-IR and ^1^H-NMR data presented above. The carbon from the ketone CO group (C-12) appears around 190 ppm in the aryl-alkyl **BQS** salts **3g**–**o**; around 166 ppm in the amide and carboxylic **BQS** salts **3a**–**c**, and around 205 ppm in the in the alkyl-alkyl **BQS** salts **3d–f**. The C-11 methylene carbon atoms appear at high chemical shifts, between 66.0–59.3 ppm, in accordance with the electron-withdrawing effect of the positive α–endocyclic nitrogen atom from benzo[f]quinoline ring and with the structure of the ketone carbonyl group (alkyl or aryl or amide/aliphatic ester). The C-2 and C-4 atoms appear around 148 ppm and 143 ppm, respectively, in accordance with their position in the pyridine ring (α- or γ- endocyclic carbons). The remaining signals in the FT-IR, ^1^H-NMR and ^13^C-NMR spectra are also in accordance with the proposed structures.

### 2.2. Antimicrobial Assay

The sensitivity of the microorganisms to the substances under investigation, salts **BQS** and cycloadducts **PBQC**, was assessed based on the diameter of the inhibition zone, using the Kirby-Bauer agar disk diffusion method, adopted by the Clinical & Laboratory Standards Institute (CLSI M07-A11, 2018) [30]. The method uses Mueller Hinton nutrient agar medium for antibacterial tests and Sabouraud nutrient agar medium for antifungal tests. The disk diffusion test provides a number of advantages, such as simplicity of the method, low cost, large number of organisms and antimicrobial agents that can be tested, and ease of interpreting the resulting data [30,31]. The in vitro antibacterial activity was evaluated against the Gram-positive bacterium *Staphylococcus aureus* ATCC 25923 and the Gram-negative *Escherichia coli* ATCC 25922. The in vitro antifungal activity was evaluated against the fungus *Candida albicans* ATCC 10231. Penicillin (10 IU), carbenicillin (100 µg/mL) and nystatin (500,000 IU) were used as positive control (C+) for *Staphylococcus aureus*, *Escherichia coli* and *Candida albicans*, respectively. As negative control (C−) sterile filter paper disks (with no antimicrobial compounds) were used. The obtained results were expressed as diameters of inhibition zones (mm). The larger the diameter of the inhibition zones is, the more active the compounds are as antimicrobials and antifungals. The obtained results are listed in Table 2 (and Figures S1–S3, see the Supporting Material for details).

The data presented in Table 2 and Figures S1–S3 reveal interesting data concerning the biological properties of salts **BQS** and cycloadducts **PBQC**. A first observation is the fact that the tested cycloadducts **PBQC** did not present any antibacterial and antifungal activity and for this reason we did not continue the assays for this category of benzo[f]quinoline derivatives. On the other hand, the tested **BQS** salts demonstrated a certain antibacterial and antifungal activity against the tested strains. Practically all the tested the **BQS** salts manifest a powerful activity against the fungus *Candida albicans*, with eight compounds being very active with a diameter of inhibition zone up to 20 mm (with two compounds, **3e** and **3f**, being remarkably active with inhibition zone diameters in the range of 30 mm) and higher that the control drug nystatin. The remaining other **BQS** salts are also active against *C. albicans*, having a diameter of inhibition zone between 15 mm (for **3k**) and 19 mm (for **3m**). Against the Gram positive *Staphylococcus aureus* bacterial strain, five **BQS** salts are very active, having an inhibition zone diameter of up to 20 mm (**3c**, **3f**, **3h**, **3i**, **3n**), while the remaining other **BQS** salts are active (with an inhibition zone diameter between 15 mm (for **3j**, **3k**, **3l**) and 19.5 mm (for **3b**)]. Against Gram negative *Escherichia coli* bacteria, the **BQS** salts have a moderate activity, six **BQS** salts being active, with a diameter of inhibition zone between 15.5 mm (for **3f**) and 18.5 mm (for **3c**).

In the next step of the antimicrobial assays, the minimum inhibitory concentration (MIC) of the ten most active **BQS** salts (namely **3b**–**e**, **3g**–**k**, **3n**) were determined, using the standardized broth microdilution assay procedure [32,33,34,35]. The resulting MIC value is defined as the lowest concentration of the antimicrobial **BQS** salts under investigation, which prevents visible growth of the tested microorganism. The obtained results are listed in Table 3.

**Figure 1:**
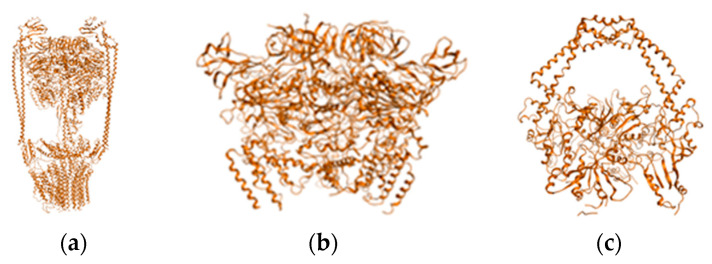
PDB structures of targeted molecules represented as ribbons in gold orange (atoms are not displayed): (**a**) ATP synthase structure; (**b**) ATP synthase outer membrane region; (**c**) Topoisomerase II.

**Figure 2:**
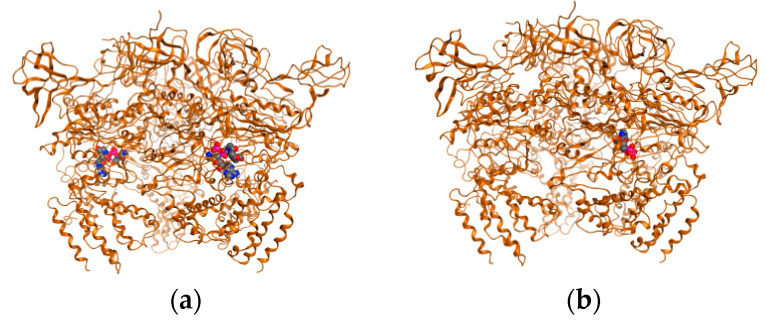
ATP synthase with ligands: (**a**) “*native*”, crystallographic determined structure; (**b**) redocked ligand (DNA string).

**Figure 3:**
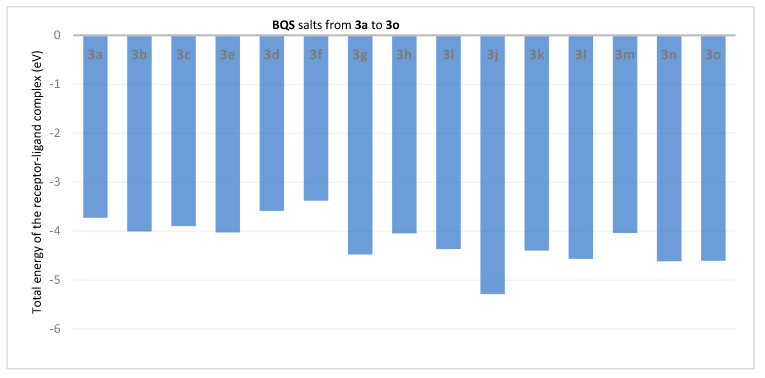
Total energy (in eV) of the receptor-ligand in **BQS 3a**–**o** salts derivative complexes with ATP synthase.

Compared with the control drug, the data from Table 3 reveal that **BQS** salt **3i** is active to a low concentration, having a MIC of 30.4 × 10^−4^ µg/mL in the case of *Staphylococcus aureus*, 15.2 × 10^−4^ µg/mL in the case of *Escherichia coli* and 575 × 10^−4^ µg/mL in the case of *Candida albicans*. Significant results were also obtained for the **BQS** salts **3n** (with a MIC of 975 × 10^−4^ µg/mL for *S. aureus* and 0.195 µg/mL for *E. coli* and *C. albicans*), **3h** and **3g** (with a MIC in the range of 0.195 µg/mL for all germs). The diameter of inhibition zone data presented in Table 2, MIC data in Table 3 and Figure 1, Figure 2 and Figure 3 reveal some interesting observations and correlations between the compound structures and their antimicrobial activity. The **BQS** salts **3a**–**o** have an excellent quasi-nonselective antifungal activity against the fungus *C. albicans*, a very good antibacterial activity against the Gram positive germ *S. aureus* and are less active against the Gram negative germ *E. coli*. The antifungal activity is significantly more pronounced in the aliphatic series **3a**–**f** compared with the aromatic one of compounds **3g**–**o**, which demonstrates a certain influence on activity of the aliphatic substituent of carbonyl group. In the aromatic series **3g**–**o**, compounds **3n** [Y = -C_6_H_4_(Cl)_p_] and **3i** [Y = -C_6_H_4_(OMe)_p_] have the highest antifungal activity, which also indicates an influence of the substituent (chlorine or methoxy) on the *para* position of the phenyl ring. The same SAR considerations are revealed by the results obtained in the antibacterial assay against the bacteria *S. aureus* and *E. coli*. Finally, if we compare the two series of tested compounds, salts **BQS 3** and cycloadducts **PBQC 4**, we may notice that only salts **BQS 3** have antimicrobial activity while the cycloadducts **PBQC 4** are inactive, which means that in order to have antimicrobial activity it is better to have a mobile substituent linked on the nitrogen atom instead of a new fused cycle.

### 2.3. Molecular Docking

During the last decades molecular docking became an important tool in medicinal chemistry, furnishing relevant information concerning binding sites, binding energy and Absorption, Distribution, Metabolism, Excretion, Toxicity (ADMET) properties. As a result, we decided to perform a computational (in silico) study concerning these parameters for the salts **BQS 3**. The chemical space was also screened for similar compounds with remarkable bioactivity and ADMET and drug-like properties. Bioactivity is studied with the aid of a computational build = receptor-ligand system. The complex is characterized by its energy, hydrogen bonds, and steric constraints. The targets (binding sites) used for this class of compounds were retrieved from the literature (mainly the work of Hunter et al. [36] and Xavier et al. [37]) and using an online service that suggests ligand predilection for a specific target [38] and are ATP synthase and topoisomerase II (TOPO II). Target structures were retrieved from the PDB database: **6WLZ** for ATP synthase [39] and **3KSB** for TOPO II [40]. The PDB structures were energetically minimized, charges corrected, names corrected, potential energy recomputed. Cofactors and Aa chains were kept, and ligands and water molecules were removed. **BQS 3** salts were introduced computationally as SDF. Files that were energetically minimized and charges corrected. Binding site coordinates (Å) were retrieved from the literature and from an algorithm based on expanded Van der Walls charges [41]. The maximum number of cavities which was set to be detected was 5, corresponding to the binding site molecular theory [42]. The cavity with the most significant volume (Å^3^) was chosen for each target (see Supporting Materials for details).

First, the two sets of compounds were docked against the two molecular targets. MOE 2009 software and its methodology were used in the docking procedure [43,44,45]. Cartesian coordinates for the two binding sites are as follows: ATP synthase: x45.94 Å, y46.91 Å, z198.20 Å; TOPO II: x-16.33 Å, y43.11 Å, z-34.50 Å. For computational and action mechanism reasons, only protein corona of ATP synthase was used in building the in-silico system (Figure 1).

Docking was validated by redocking the ligands present in PDB target structures. For this step ligands were cut from the crystallographic obtained structures converted to SDF files, minimized and charges corrected. Ligands were then docked on to the free ligand PDB structures. Results are depicted in Figure 2.

**Figure 4:**
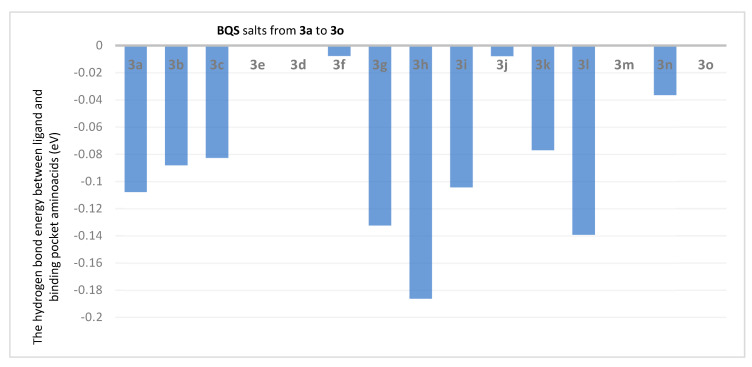
The hydrogen bond energy between ligand and binding pocket aminoacids (in eV) in **BQS 3a**–**o** salt derivative complexes with ATP synthase.

Figure 3 and Figure 4, describe the interaction of each **BQS** salt **3a**–**o** with ATP synthase in the formed complex, in terms of total energies of the complexes (E_T_) and hydrogen bond energy (E_H_).

Analysis of the data from Figure 3 reveals that the compounds **3j** (E_T_ = −5.39 eV), **3g** (E_T_ = −4.48 eV), **3i** (E_T_ = −4.37 eV), **3l** (E_T_ = −4.57 eV) and **3n** (E_T_ = −4.62 eV) show the lowest energies in complex with ATP synthase. The data from Figure 4 reveals that the compounds **3h** (E_H_ = −0.1863 eV), **3g** (E_H_ = −0.1325 eV), **3i** (E_H_ = −0.1044 eV) and **3l** (E_H_ = −0.1393 eV) show the lowest hydrogen bond energy of the ATP synthase complex; compounds **3e**, **3d**, **3m**, and **3o** don’t form hydrogen bonds.

**Figure 5:**
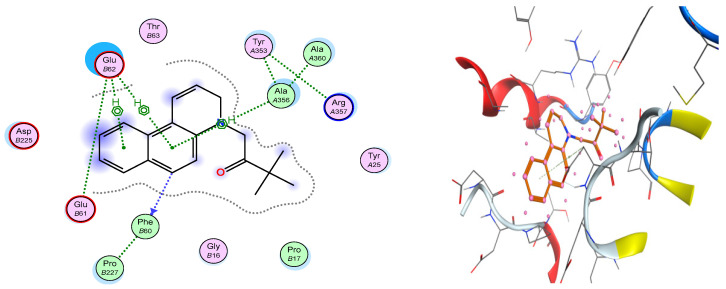
Compound **3f** is represented in complex with ATP synthase as sticks. Surrounding aminoacids at the binding site are represented as ribbons colored by chain type. Also, a schematic view of the binding site pocket is represented.

In Figure 5 is presented the complex with ATP synthase of **BQS** salt **3f**. In the binding site pocket we may notice two powerful H-π interactions between the two benzene rings from the benzo[f]quinoline moiety and the aminoacid Glu 62. Another H-π interaction is formed between Ala 356 and the benzene from the center of the aromatic core. Also, Ala 356 has interresidue contacts with Tyr 353 and Arg 357. A benzene carbon donates electrons to Phe 60, which has an inter-residue contact with Pro 227. All these interactions are stabilizing the **BQS** salt **3f**—ATP synthase complex. 

**Figure 6:**
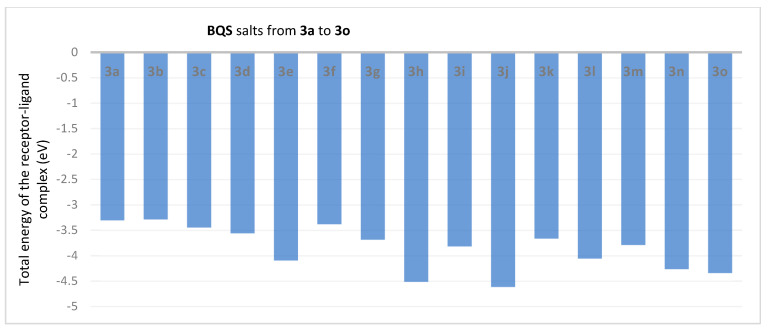
**BQS 3a–o** salts in complex with TOPO II: energies of complexes.

**Figure 7:**
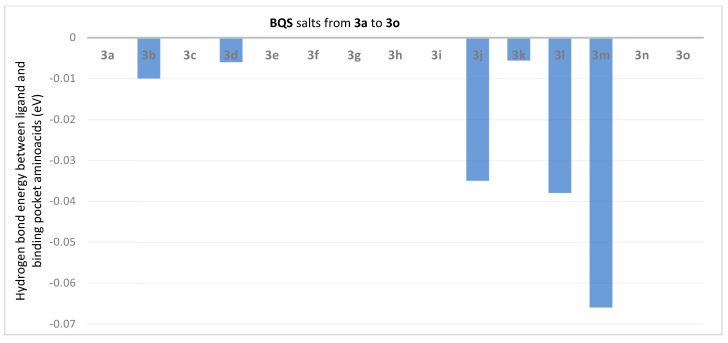
The hydrogen bond energy (in eV) between ligand and binding pocket aminoacids in **BQS 3a–o** salts derivates complexes with TOPO II.

In the next step, a similar protocol was followed to study the interactions of **BQS** salts with TOPO II. Figure 6 and Figure 7, describe the interaction of each **BQS 3a**–**o** salt with TOPO II in the formed complex, in terms of total energies of the complexes (E_T_) and hydrogen bond energy (E_H_).

Analysis of the data from Figure 6 reveal that the compounds **3j** (E_T_ = −4.617 eV), **3h** (E_T_ = −4.517 eV), **3e** (E_T_ = −4.097 eV) and **3l** (E_T_ = −4.059 eV) show the lowest energies in complex with TOPO II. The data from Figure 7 reveal that the compounds **3m** (E_H_ = −0.066 eV), **3l** (E_H_ = −0.038 eV) and **3j** (E_H_ = −0.035 eV) show the lowest hydrogen bond energy of the TOPO II complex; compounds **3a**, **3c**, **3e**, **3f**, **3g**, **3h**, **3i**, **3n** and **3o** don’t form hydrogen bonds.

**Figure 8:**
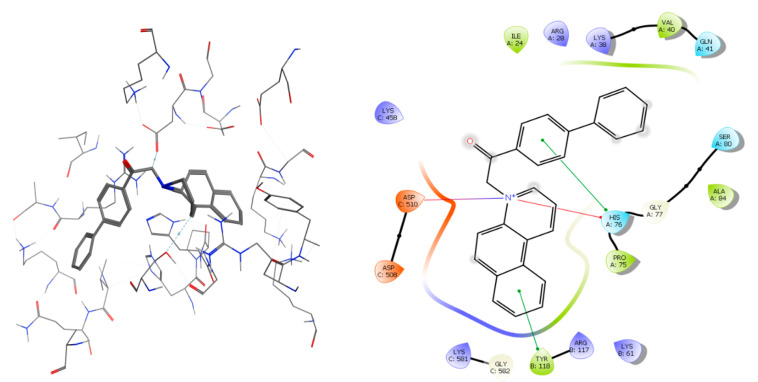
Compound **3j** is represented in complex with TOPO II as sticks. Surrounding aminoacids at the binding site are represented as ribbons colored by chain type. Also, a schematic view of the binding pocket is represented.

From the obtained data we may notice that salts **3j** and **3n** have the best-fit in complex with TOPO II (in Figure 8 the complex of salt **3j** is presented). In the binding pocket of TOPO II, a hydrogen bond is formed between the nitrogen atom of the benzo[f]quinoline ring and aminoacid ASP-510, stabilizing the salt BQS **3j**—TOPO II complex. We also noticed that TYR 118 and His 76 interact with the aromatic rings. Also, a π bond is observed between His 76 (aromatic) and the positive nitrogen atom. 

**Figure 9:**
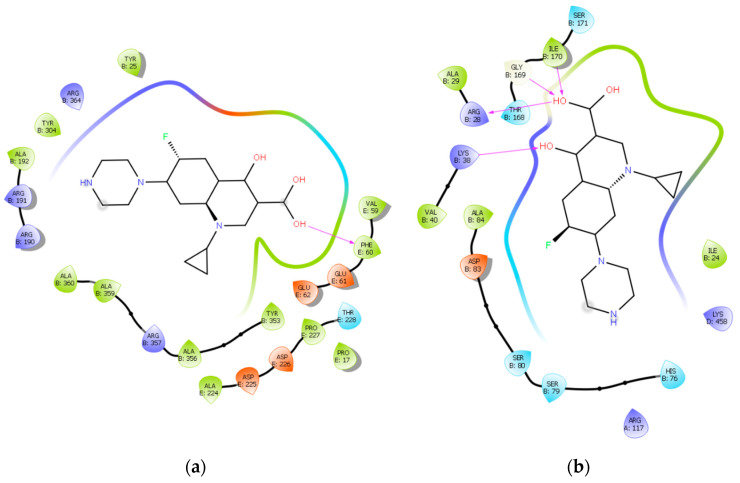
Ciprofloxacin in complex with: (**a**) ATP synthase where hydrogen bond is formed between an OH group and Phe 60; (**b**) TOPO II, where hydrogen bonds are formed between OH groups and Lys 38, Arg 28, Gly 169, and Ile 170, respectively.

Ciprofloxacin, the control molecule, when docked with ATP synthase, shows a total energy of the complex of −2.59 eV and a hydrogen bond energy of −8.31 eV. In a complex with TOPO II, ciprofloxacin shows a total energy of −2.56 eV and a hydrogen bonding energy of −2.037 eV (Figure 9). 

**Figure 10:**
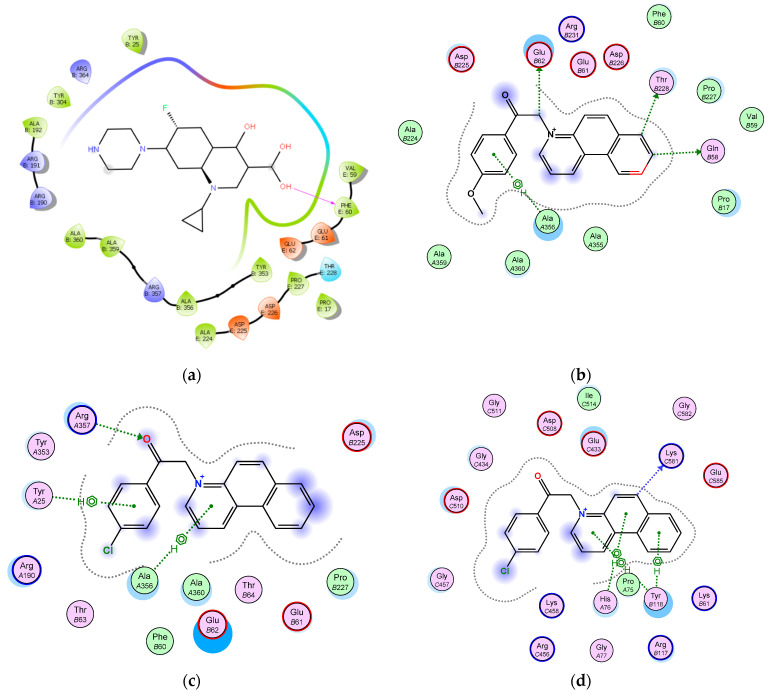
**3i** and **3n** in complex with ATP synthase and TOPO II in their binding pockets: (**a**) **3i** in complex with ATP synthase arene hydrogen bonds are formed between aromatic core and Ala 366, Glu 62, Thr 228, and Gln 58 accept electrons from aromatic and aliphatic carbons; (**b**) **3i** in complex with TOPO II, nitrogen atom interacts with Asp 508 and Glu 433, heterocyclic aromatic carbon share electrons with Asp 508 and Glu 433; (**c**) **3n** in complex with ATP synthase, arene hydrogen bonds are formed between an aromatic core and Tyr 25 and between heterocycle aromatic core and Ala 356; also keto group shares electrons with Arg 357; (**d**) **3n** in complex with TOPO II, arene hydrogen bonds are formed between each heterocyclic aromatic core and His 76, Pro 75, and Tyr 118, respectively.

The most active antimicrobial compounds **3i** and **3n** show a similar behaviour in the ATP synthase and TOPO II binding pockets as compound **3j** (Figure 10).

Lastly, regarding our computational docking study, compound **3j** displayed the best interaction energies. However, experimentally compounds **3i** and **3n** have the best bioactivities, while **3j** has only moderate antimicrobial activity. In comparison with cyprofloxacin where the aromatic centres seem to play no role, in the case of **BQS** those aromatic centres are crucial in the interaction with the active sites. In the next step, the ADME properties of **BQS** salts **3a–o** were studied (Table 4).

Analysis of the data from Table 4 reveals interesting data concerning the **BQS** salts’ ADME properties. All salt derivatives show good aqueous solubility. This is indicated by two descriptors: *QP logS* (which describes the general aqueous solubility) and *ClQP logS* (which describes the conformation-independent aqueous solubility). The optimal values of these two descriptors are in the range between −6.5 and 0.5. The data from Table 4 indicate for our **BQS** salt **3a** has good solubility. Three compounds (**3j**, **3m**, and **3n**) are exceptions being slightly out of limit.

The *QP log HERG* descriptor is a computational equivalent of zebrafish model toxicity in respect to the mode of action (MOA). This descriptor is linked with the potential K channel-blocking effect due to the electrophilic nature of drugs. The concerned values are below −5. As observed from Table 4 the majority of the synthesized compounds have predictable, convenient values, which means that our **BQS** quaternary salts **3a-o** have low toxicity profiles. The reduced toxicity it is also confirmed by the Lipinski’s rule of five and Jorgensen’s rule of three computational results. From Table 4 we can notice that only compounds **3j**, **3m**, and **3n** present a violation of Lipinski’s rule of 5 and Jorgensen’s rule of 3, respectively. According with Lipinski’s rule of five, the maximum number of violations for a computed compound is five (these five rules are: mol_MW < 500; QP log Po/w < 5; donor HB < 5; accpt. HB < 10) while for Jorgensen’s rule of three, the maximum number of violations for a computed compound is three (QP log s > −5.7; QP PCaco > 22 nm/s; primary metabolites < 7). As a result, all our **BQS** salts **3a-o** have excellent drug-like properties. However, in vitro testing should be performed in order to confirm these theoretical results.

The *QP PCaco* descriptor predicts permeability at the gut drug barrier. Predictions are for non-active transport. Values below 25 describe a low permeability while values higher than 500 are characteristic of excellent permeability. All described compounds show poor predicted gut barrier permeability. 

*QP Log BB* descriptor predicts brain-blood partition coefficient for oral delivery. An optimal interval is between descriptor values of −3.0 to 1.2. All compounds are off the limit, meaning that potentially they may have poor blood-brain partition coefficients when administered orally.

*QP PMDCK* is a descriptor designed to simulate MDCK cell permeability, considered to be a good mimic of the blood-brain barrier. Values below 25 describe a poor permeability while values higher than 500 describe an excellent permeability. The data from Table 4 indicate that all **BQS** salts **3a**–**o** has excellent blood—brain barrier permeability. 

*QP log KP* is a descriptor that predicts skin permeability, with an optimal interval between −8.0 and −1.0. The data from Table 4 indicate that the compounds might not have good skin absorption.

Predicted chemical reactivity (descriptor *Metab.*) shows very high values for our **BQS** salts, suggesting a negative behavior as leads (the optimal values for *Metab.* descriptor are range between 1 to −8). 

Interactions of compounds with serum albumin were explored computationally using the *QP log Khsa* descriptor. The optimal values for *QP log Khsa* descriptor are in the range between −1.5 to 15. The data from Table 4 indicate that all **BQS** salts **3a**–**o** have a high potential of interacting with human serum albumin. 

*%Ab-sorbtion* is a descriptor that predicts human oral absorption on a 0 to 100% scale. Values below 25% describe a poor oral absorption while value higher than 80% describe an excellent oral absorption. The data from Table 4 indicate that all **BQS** salts **3a**–**o** have values higher than 80%, which suggest excellent oral absorption.

## 3. Materials and Methods 

### 3.1. General Information

Reagents and solvents were purchased from commercial sources and used without further purification. The melting points (uncorrected) of compounds were recorded in open capillary tubes using a MEL-TEMP Electrothermal apparatus (Barnstead International, Dubuque, IA, USA). The nuclear magnetic resonance spectra were recorded on an Avance III 500 MHz spectrometer (Bruker, Vienna, Austria) operating at 500 MHz for ^1^H and 125 MHz for ^13^C. Chemical shifts were reported in delta (δ) units (ppm), relative to the residual peak of solvent (ref: DMSO, ^1^H: 2.50 ppm; ^13^C: 39.52 ppm), and coupling constants (*J*) in Hz. In the NMR spectra the multiplicity of signals was indicated using the abbreviations s = singlet, bs = broad singlet, d = doublet, ad = apparent doublet, add = apparent doublet of doublets, t = triplet, at = apparent triplet, td = triplet of doublets, atd = apparent triplet of doublets, q = quartet, m = multiplet. The IR spectra were recorded using a VERTEX 70 FTIR spectrometer (Bruker, Vienna, Austria) equipped with an ATR module. Thin layer chromatography (TLC) was performed on commercial silica gel plates (silica gel 60 F_254_ plates, Merck, Darmstadt, Germany), the visualization being done using a UV lamp (λ_max_ = 254 or 365 nm). The microanalysis results were in satisfactory agreement (C, ±0.15; H, ±0.10; N, ±0.30) with the calculated values. Compound **3i** was already reported in the literature, and its spectral data were in agreement with the reported data [29].

### 3.2. General Procedure for the Synthesis of Benzo[f]quinoline Salts **3a–o**

**Scheme 3:**
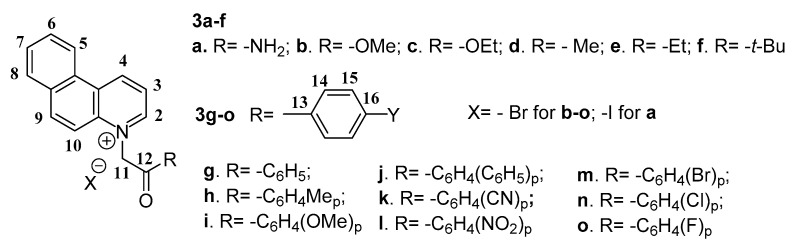
Chemical Structures of compounds **3a**–**o**.

A solution of halogenated derivatives **2a**–**f** with aliphatic chains and increased reactivity (1.1 mmol) or with aromatic skeletons (compounds **2g**–**o**) (1.5 mmol) in 15 mL of acetone was added dropwise in a solution of benzo[f]quinoline (**1**, 1 mmol) in 15 mL of acetone. The obtained mixture was heated at reflux during 16 h, and then stirred for 12 h at room temperature. The precipitated quaternary salts **3a**–**o** (Scheme 3) were filtered off, washed two times with 5 mL of acetone, and then dried under vacuum. No further purification was required. 

#### 3.2.1. 1-(2-Amino-2-oxoethyl)benzo[f]quinolin-1-ium iodide (**3a**)

Yellowish green powder; yield: 84%; m.p. 238–240 °C; IR, ν_max_ 3335, 3134, 3037, 2966, 1681, 1597, 1412, 1344 cm^−1^; ^1^H-NMR (DMSO-*d*_6_) δ 10.18 (1H, d, *J* = 8.5 Hz, H-4), 9.47 (1H, d, *J* = 6.0 Hz, H-2), 9.10 (1H, d, *J* = 8.5 Hz, H-5), 8.68 (1H, d, *J* = 9.5 Hz, H-9), 8.40 (1H, td, *J* = 8.5 Hz, *J* = 6.0 Hz, H-3), 8.28 (1H, d, *J* = 8.0 Hz, H-8), 8.20 (1H, bs, NH), 8.14 (1H, d, *J* = 9.5 Hz, H-10), 8.01 (1H, t, *J* = 7.5 Hz, H-6), 7.95 (1H, t, *J* = 7.5 Hz, H-7), 7.88 (1H, bs, NH), 5.91 (2H, s, CH_2_); ^13^C-NMR (DMSO-*d*_6_) δ 166.0 (C = O amido), 148.5 (C-2), 142.1 (C-4), 139.6 (C-10a), 138.0 (C-9), 130.7 (C-8a), 130.1 (C-6), 130.0 (C-7), 129.4 (C-8), 127.8 (C-4a, C-4b), 124.2 (C-5), 122.6 (C-3), 115.7 (C-10), 59.3 (CH_2_); Anal. Calcd. for C_15_H_13_IN_2_O C, 49.47; H, 3.60; N, 7.69. Found C, 49.37; H, 3.70; N, 7.49.

#### 3.2.2. 1-(2-Methoxy-2-oxoethyl)benzo[f]quinolin-1-ium bromide (**3b**)

Yellowish powder; yield: 70%; m.p. 149–153 °C; IR (KBr), ν_max_ 3029, 2869, 1743, 1600, 1431, 1232, 1068 cm^−1^; ^1^H-NMR (400 MHz, DMSO-*d*_6_) δ 10.23 (1H, d, *J* = 8.8 Hz, H-4), 9.62 (1H, d, *J* = 6.0 Hz, H-2), 9.09 (1H, d, *J* = 8.0 Hz, H-5), 8.67 (1H, d, *J* = 9.6 Hz, H-9), 8.44 (1H, atd, *J* = 8.4 Hz, *J* = 6.0 Hz, H-3), 8.34 (1H, d, *J* = 10.0 Hz, H-10), 8.28 (1H, d, *J* = 7.6 Hz, H-8), 8.01 (2H, m, H-6, H-7), 6.33 (2H, s, CH_2_), 3.81 (3H, s, CH_3_); ^13^C-NMR (100 MHz, DMSO-*d*_6_) δ 166.6 (C = O ester), 148.3 (C-2), 142.9 (C-4), 139.5 (C-10a), 138.2 (C-9), 130.7 (C-8a), 130.1 (C-6), 130.0 (C-7), 129.3 (C-8), 127.8 (C-4a), 127.7 (C-4b), 124.1 (C-5), 122.7 (C-3), 115.9 (C-10), 57.9 (CH_2_), 53.2 (CH_3_); Anal. Calcd. for C_16_H_14_BrNO_2_ C, 57.85; H, 4.25; N, 4.22. Found C, 57.75; H, 4.20; N, 4.12.

#### 3.2.3. 1-(2-Ethoxy-2-oxoethyl)benzo[f]quinolin-1-ium bromide (**3c**)

Yellowish powder; yield: 73%; m.p. 125–127 °C; IR (KBr), ν_max_ 3058, 2977, 1741, 1598, 1417, 1209, 1014 cm^−1^; ^1^H-NMR (DMSO-*d*_6_) δ 10.25 (1H, d, *J* = 8.4 Hz, H-4), 9.60 (1H, d, *J* = 5.6 Hz, H-2), 9.12 (1H, d, *J* = 8.0 Hz, H-5), 8.70 (1H, d, *J* = 9.6 Hz, H-9), 8.45 (1H, atd, *J* = 8.4 Hz, *J* = 6.0 Hz, H-3), 8.32 (2H, m, H-8, H-10), 7.99 (2H, m, H-6, H-7), 6.30 (2H, s, CH_2_), 4.26 (2H, q, *J* = 7.2 Hz, CH_2_-Et), 1.26 (3H, t, *J* = 7.2 Hz, CH_3_); ^13^C-NMR (DMSO-*d*_6_) δ 166.1 (C = O ester), 148.4 (C-2), 142.8 (C-4), 139.6 (C-10a), 138.2 (C-9), 130.7 (C-8a), 130.1 (C-6), 130.0 (C-7), 129.4 (C-8), 127.8 (C-4a), 127.7 (C-4b), 124.2 (C-5), 122.7 (C-3), 115.9 (C-10), 62.4 (CH_2_-Et), 57.9 (CH_2_), 13.9 (CH_3_); Anal. Calcd. for C_17_H_16_BrNO_2_ C, 58.97; H, 4.66; N, 4.05. Found C, 58.87; H, 4.60; N, 4.15.

#### 3.2.4. 1-(2-Oxopropyl)benzo[f]quinolin-1-ium bromide (**3d**) 

Beige powder; yield: 67%; m.p. 159–160 °C; IR, ν_max_ 3022, 2855, 1730, 1597, 1511, 1411, 1341, 1173 cm^−1^; ^1^H-NMR (DMSO-*d*_6_) δ 10.20 (1H, d, *J* = 8.5 Hz, H-4), 9.39 (1H, d, *J* = 5.5 Hz, H-2), 9.12 (1H, d, *J* = 8.0 Hz, H-5), 8.64 (1H, d, *J* = 9.5 Hz, H-9), 8.44 (1H, dd, *J* = 5.5 Hz, *J* = 8.5 Hz, H-3), 8.30 (2H, m, H-8, H-10), 8.03 (1H, t, *J* = 7.5 Hz, H-6), 7.96 (1H, t, *J* = 7.5 Hz, H-7), 6.43 (2H, s, CH_2_), 2.50 (3H, s, CH_3_); ^13^C-NMR (DMSO-*d*_6_) δ 199.7 (C = O keto), 147.8 (C-2), 144.0 (C-4), 142.3 (C-10a), 139.8 (C-9), 130.8 (C-4a), 130.1 (C-7), 130.0 (C-6), 129.4 (C-8), 127.9 (C-4b), 127.8 (C-9), 124.2 (C-5), 122.7 (C-3), 116.4 (C-10), 66.0 (CH_2_), 27.6 (CH_3_); Anal. Calcd. for C_16_H_14_BrNO C, 60.78; H, 4.46; N, 4.43. Found C, 60.69; H, 4.42; N, 4.38.

#### 3.2.5. 1-(2-Oxobutyl)benzo[f]quinolin-1-ium bromide (**3e**)

Yellow powder; yield: 71%; m.p. 165–166 °C; IR, ν_max_ 3052, 2964, 1723, 1599, 1413, 1340, 1106 cm^−1^; ^1^H-NMR (DMSO-*d*_6_) δ 10.21 (1H, d, *J* = 8.5 Hz, H-4), 9.40 (1H, d, *J* = 6.0 Hz, H-2), 9.12 (1H, d, *J* = 8.5 Hz, H-5), 8.63 (1H, d, *J* = 10.0 Hz, H-9), 8.42 (1H, dd, *J* = 6.0 Hz, *J* = 8.5 Hz, H-3), 8.30 (2H, m, H-8, H-10), 8.02 (1H, t, *J* = 7.5 Hz, H-6), 7.96 (1H, t, *J* = 7.5 Hz, H-7), 6.42 (2H, s, CH_2_), 2.93 (2H, q, *J* = 7.5 Hz, CH_2_-Et), 1.06 (3H, t, *J* = 7.5 Hz, CH_3_-Et); ^13^C-NMR (DMSO-*d*_6_) δ 202.3 (C = O keto), 147.8 (C-2), 142.2 (C-4), 139.8 (C-10a), 137.9 (C-9), 130.8 (C-4a), 130.1 (C-7), 130.0 (C-6), 129.4 (C-8), 127.9 (C-4b), 127.8 (C-9), 124.2 (C-5), 122.7 (C-3), 116.3 (C-10), 65.3 (CH_2_), 32.8 (CH_2_-Et), 6.9 (CH_3_-Et); Anal. Calcd. for C_17_H_16_BrNO C, 61.83; H, 4.88; N, 4.24. Found C, 61.73; H, 4.81; N, 4.17.

#### 3.2.6. 1-(3,3-Dimethyl-2-oxobutyl)benzo[f]quinolin-1-ium bromide (**3f**)

Yellow powder; yield: 89%; m.p. 129–130 °C; IR, ν_max_ 3051, 2942, 1708, 1578, 1414, 1342, 1057 cm^−1^; ^1^H-NMR (DMSO-*d*_6_) δ 10.24 (1H, d, *J* = 8.5 Hz, H-4), 9.46 (1H, d, *J* = 6.0 Hz, H-2), 9.15 (1H, d, *J* = 8.5 Hz, H-5), 8.68 (1H, d, *J* = 9.5 Hz, H-9), 8.45 (1H, dd, *J* = 6.0 Hz, *J* = 8.5 Hz, H-3), 8.30 (1H, d, *J* = 7.5 Hz, H-8), 8.08 (1H, d, *J* = 9.5 Hz, H-10), 8.04 (1H, t, *J* = 7.5 Hz, H-6), 7.98 (1H, t, *J* = 7.5 Hz, H-7), 6.68 (2H, s, CH_2_), 1.36 (9H, s, 3 × CH_3_-^t^Bu); ^13^C-NMR (DMSO-*d*_6_) δ 207.0 (C = O keto), 148.2 (C-2), 142.3 (C-4), 139.6 (C-10a), 138.1 (C-9), 130.8 (C-4a), 130.2 (C-7), 130.1 (C-6), 129.4 (C-8), 128.0 (C-4b), 127.9 (C-9), 124.3 (C-5), 122.8 (C-3), 115.7 (C-10), 63.0 (CH_2_), 43.4 (C(CH_3_)_3_-^t^Bu), 25.7 (3 × CH_3_-^t^Bu); Anal. Calcd. for C_19_H_20_BrNO C, 63.70; H, 5.63; N, 3.91. Found C, 63.59; H, 5.56; N, 3.86.

#### 3.2.7. 1-(Phenacyl)benzo[f]quinolin-1-ium bromide (**3g**)

Cream-colored powder; yield: 61%; m.p. 234–236 °C; IR, ν_max_ 2989, 2898, 1682, 1577, 1343, 1222, 1065 cm^−1^; ^1^H-NMR (500 MHz, DMSO-*d*_6_) δ 10.20 (1H, d, *J* = 8.5 Hz, H-4), 9.45 (1H, d, *J* = 5.5 Hz, H-2), 9.11 (1H, d, *J* = 8.5 Hz, H-5), 8.56 (1H, d, *J* = 10 Hz, H-9), 8.39 (1H, dd, *J* = 8.5 Hz, *J* = 6.0 Hz, H-3), 8.23 (2H, d, *J* = 9.5 Hz, *J* = 7.5 Hz, H-8, H-10), 8.17 (2H, d, *J* = 7.0 Hz, 2 × H-14), 8.02 (1H, atd, *J* = 8.0 Hz, *J* = 7.0 Hz, H-6), 7.95 (1H, t, *J* = 8.0 Hz, *J* = 7.0 Hz, H-7), 7.80 (1H, d, *J* = 7.5 Hz, H-16), 7.67 (2H, t, *J* = 8.0 Hz, 2 × H-15), 7.05 (2H, s, CH_2_); ^13^C-NMR (125 MHz, DMSO-*d*_6_) δ 190.6 (C = O keto), 148.2 (C-2), 142.5 (C-4), 140.3 (C-10a), 138.4 (C-9), 135.0 (C-16), 133.6 (C-13), 131.0 (C-8a), 130.3 (C-7), 130.2 (C-6), 129.5 (C-8), 129.1 (2 × C-15), 128.7 (2 × C-14), 128.3 (C-4a), 128.1 (C-4b), 124.2 (C-5), 122.9 (C-3), 116.1 (C-10), 63.9 (CH_2_); Anal. Calcd. for C_21_H_16_BrNO C, 66.68; H, 4.26; N, 3.70. Found C, 66.78; H, 4.16; N, 3.65.

#### 3.2.8. 1-(4-Methylphenacyl)benzo[f]quinolin-1-ium bromide (**3h**)

Yellowish powder; yield: 57%; m.p. 220–224 °C; IR, ν_max_ 3027, 2946, 1677, 1600, 1344, 1231, 1185 cm^−1^; ^1^H-NMR (DMSO-*d*_6_) δ 10.25 (1H, d, *J* = 8.5 Hz, H-4), 9.55 (1H, d, *J* = 5.5 Hz, H-2), 9.15 (1H, d, *J* = 8.0 Hz, H-5), 8.61 (1H, d, *J* = 9.5 Hz, H-9), 8.46 (1H, atd, *J* = 8.5 Hz, *J* = 6.0 Hz, H-3), 8.28 (2H, m, H-8, H-10), 8.08 (2H, d, *J* = 8.0 Hz, 2 × H-14), 8.02 (1H, t, *J* = 8.0 Hz, *J* = 7.0 Hz, H-6), 7.96 (1H, t, *J* = 7.5 Hz, H-7), 7.50 (2H, d, *J* = 8.0 Hz, 2 × H-15), 7.12 (2H, s, CH_2_), 2.46 (3H, s, CH_3_); ^13^C-NMR (DMSO-*d*_6_) δ 190.2 (C = O keto), 148.2 (C-2), 145.6 (C-16), 142.4 (C-4), 140.0 (C-10a), 138.0 (C-9), 131.1 (C-13), 130.8 (C-8a), 130.1 (C-7), 130.0 (C-6), 129.5 (2 × C-15), 129.4 (C-8), 128.8 (2 × C-14), 127.9 (C-4a), 127.8 (C-4b), 124.2 (C-5), 122.8 (C-3), 116.3 (C-10), 63.8 (CH_2_), 21.4 (CH_3_); Anal. Calcd. for C_22_H_18_BrNO C, 67.36; H, 4.62; N, 3.57. Found C, 67.47; H, 4.52; N, 3.47.

#### 3.2.9. 1-(4-Methoxyphenacyl)benzo[f]quinolin-1-ium bromide (**3i**)

Yellowish powder; yield: 70%; m.p. 139–142 °C; IR, ν_max_ 2968, 2900, 1673, 1598, 1418, 1344, 1237, 1179, 1021 cm^−1^; ^1^H-NMR (DMSO-*d*_6_) δ 10.25 (1H, d, *J* = 8.5 Hz, H-4), 9.52 (1H, d, *J* = 5.5 Hz, H-2), 9.15 (1H, d, *J* = 8.0 Hz, H-5), 8.62 (1H, d, *J* = 9.5 Hz, H-9), 8.46 (1H, t, *J* = 8.0 Hz, *J* = 6.5 Hz, H-3), 8.27 (2H, at, *J* = 8.0 Hz, H-8, H-10), 8.15 (2H, d, *J* = 8.5 Hz, 2 × H-14), 8.03 (1H, t, *J* = 8.0 Hz, *J* = 7.0 Hz, H-6), 7.96 (1H, t, *J* = 7.5 Hz, *J* = 7.0 Hz, H-7), 7.22 (2H, d, *J* = 8.5 Hz, 2 × H-15), 7.08 (2H, s, CH_2_), 3.92 (3H, s, CH_3_); ^13^C-NMR (DMSO-*d*_6_) δ 188.9 (C = O keto), 164.4 (C-16), 148.2 (C-2), 142.4 (C-4), 140.0 (C-10a), 138.0 (C-9), 131.2 (2 × C-14), 130.8 (C-8a), 130.1 (C-7), 130.0 (C-6), 129.4 (C-8), 127.9 (C-4a), 127.8 (C-4b), 126.4 (C-13), 124.2 (C-5), 122.8 (C-3), 116.3 (C-10), 114.3 (2 × C-15), 63.5 (CH_2_), 55.9 (CH_3_); Anal. Calcd. for C_22_H_18_BrNO_2_ C, 64.72; H, 4.44; N, 3.43. Found C, 64.62; H, 4.40; N, 3.63.

#### 3.2.10. 1-(4-Phenylphenacyl)benzo[f]quinolin-1-ium bromide (**3j**)

Yellowish powder; yield: 80%; m.p. 242–245 °C; IR, ν_max_ 3016, 2895, 1689, 1601, 1339, 1225 cm^−1^; ^1^H-NMR (DMSO-*d*_6_) δ 10.28 (1H, d, *J* = 8.5 Hz, H-4), 9.56 (1H, add, *J* = 6.5 Hz, H-2), 9.17 (1H, d, *J* = 8.5 Hz, H-5), 8.64 (1H, d, *J* = 10 Hz, H-9), 8.48 (1H, dd, *J* = 8.5 Hz, *J* = 6.0 Hz, H-3), 8.36 (1H, d, *J* = 10.0 Hz, H-10), 8.30 (1H, d, *J* = 7.5 Hz, H-8), 8.26 (2H, d, *J* = 8.5 Hz, 2 × H-14), 8.03 (3H, m, 2 × H-15, H-6), 7.97 (1H, atd, *J* = 8.0 Hz, *J* = 7.5 Hz, H-7), 7.84 (2H, d, *J* = 8.5 Hz, 2 × H-18), 7.55 (2H, t, *J* = 8.0 Hz, *J* = 7.0 Hz, 2 × H-19), 7.48 (1H, atd, *J* = 8.5 Hz, *J* = 7.0 Hz, H-20), 7.18 (2H, s, CH_2_); ^13^C-NMR (DMSO-*d*_6_) δ 190.3 (C = O keto), 148.2 (C-2), 146.0 (C-16), 142.5 (C-4), 140.1 (C-10a), 138.5 (C-17), 138.9 (C-9), 132.4 (C-13), 130.8 (C-8a), 130.1 (C-6), 130.0 (C-7), 129.4 (2 × C-14, C-8), 129.2 (2 × C-19), 128.8 (C-20), 127.9 (C-4a), 127.8 (C-4b), 127.1 (2 × C-18, 2 × C-15), 124.3 (C-5), 122.8 (C-3), 116.4 (C-10), 63.9 (CH_2_); Anal. Calcd. for C_27_H_20_BrNO C, 71.37; H, 4.44; N, 3.08. Found C, 71.47; H, 4.34; N, 3.18.

#### 3.2.11. 1-(4-Cyanophenacyl)benzo[f]quinolin-1-ium bromide (**3k**)

Yellow powder; yield: 85%; m.p. 199–202 °C; IR, ν_max_ 3015, 2952, 2232, 1706, 1401, 1340, 1214 cm^−1^; ^1^H-NMR (DMSO-*d*_6_) δ 10.28 (1H, d, *J* = 8.5 Hz, H-4), 9.51 (1H, d, *J* = 6.0 Hz, H-2), 9.17 (1H, d, *J* = 8.5 Hz, H-5), 8.64 (1H, d, *J* = 9.5 Hz, H-9), 8.48 (1H, atd, *J* = 8.5 Hz, *J* = 6.0 Hz, H-3), 8.41 (1H, d, *J* = 9.5 Hz, H-10), 8.31 (3H, t, *J* = 8.5 Hz, H-8, 2 × H-14), 8.20 (2H, d, *J* = 8.5 Hz, 2 × H-15), 8.04 (1H, t, *J* = 8.0 Hz, *J* = 7.5 Hz, H-6), 7.98 (1H, t, *J* = 8.0 Hz, *J* = 7.0 Hz, H-7), 7.15 (2H, s, CH_2_); ^13^C-NMR (DMSO-*d*_6_) δ 190.3 (C = O keto), 148.2 (C-2), 142.6 (C-4), 140.2 (C-10a), 138.1 (C-9), 136.9 (C-13), 132.9 (2 × C-15), 130.8 (C-8a), 130.1 (C-7, C-6), 129.4 (C-8), 129.3 (2 × C-14), 127.9 (C-4a), 127.8 (C-4b), 124.3 (C-5), 122.8 (C-3), 118.0 (CN), 116.4 (C-10), 116.3 (C-16), 64.1 (CH_2_); Anal. Calcd. for C_22_H_15_BrN_2_O C, 65.52; H, 3.75; N, 6.95. Found C, 65.62; H, 3.70; N, 6.85.

#### 3.2.12. 1-(4-Nitrophenacyl)benzo[f]quinolin-1-ium bromide (**3l**)

Yellowish powder; yield: 74%; m.p. 156–158 °C; IR, ν_max_ 3023, 2963, 1717, 1599, 1520, 1349, 1217 cm^−1^; ^1^H-NMR (DMSO-*d*_6_) δ 10.29 (1H, d, *J* = 8.5 Hz, H-4), 9.52 (1H, d, *J* = 5.5 Hz, H-2), 9.17 (1H, d, *J* = 8.5 Hz, H-5), 8.65 (1H, d, *J* = 9.5 Hz, H-9), 8.52 (2H, d, *J* = 9.0 Hz, 2 × H-15), 8.48 (1H, at, *J* = 8.5 Hz, *J* = 6.0 Hz, H-3), 8.43 (1H, d, *J* = 10.0 Hz, H-10), 8.40 (2H, d, *J* = 8.5 Hz, 2 × H-14), 8.13 (1H, d, *J* = 7.5 Hz, H-8), 8.50 (1H, t, *J* = 8.0 Hz, *J* = 7.0 Hz, H-6), 7.98 (1H, t, *J* = 7.5 Hz, *J* = 7.0 Hz, H-7), 7.18 (2H, s, CH_2_); ^13^C-NMR (DMSO-*d*_6_) δ 190.1 (C = O keto), 150.6 (C-16), 148.2 (C-2), 142.7 (C-4), 140.2 (C-10a), 138.3 (C-13), 138.1 (C-9), 130.8 (C-8a), 130.2 (2 × C-14), 130.1 (C-7, C-6), 129.4 (C-8), 128.0 (C-4a), 127.8 (C-4b), 124.2 (C-5), 124.0 (2 × C-15), 122.8 (C-3), 116.4 (C-10), 64.1 (CH_2_); Anal. Calcd. for C_21_H_15_BrN_2_O_3_ C, 59.59; H, 3.57; N, 6.62. Found C, 59.49; H, 3.50; N, 6.52.

#### 3.2.13. 1-(4-Bromophenacyl)benzo[f]quinolin-1-ium bromide (**3m**)

Cream-colored powder; yield: 80%; m.p. 229–232 °C; IR, ν_max_ 3018, 2911, 1696, 1583, 1347, 1223 cm^−1^; ^1^H-NMR (DMSO-*d*_6_) δ 10.27 (1H, d, *J* = 9.0 Hz, H-4), 9.52 (1H, d, *J* = 6.0 Hz, H-2), 9.16 (1H, d, *J* = 8.5 Hz, H-5), 8.63 (1H, d, *J* = 10.0 Hz, H-9), 8.47 (1H, atd, *J* = 8.5 Hz, *J* = 6.0 Hz, H-3), 8.35 (1H, d, *J* = 9.5 Hz, H-10), 8.29 (1H, d, *J* = 7.5 Hz, H-8), 8.10 (2H, d, *J* = 8.5 Hz, 2 × H-14), 8.36 (1H, t, *J* = 7.5 Hz, *J* = 7.0 Hz, H-6), 7.97 (1H, t, *J* = 7.5 Hz, H-7), 7.94 (2H, d, *J* = 8.5 Hz, 2 × H-15), 7.11 (2H, s, CH_2_); ^13^C-NMR (DMSO-*d*_6_) δ 190.1 (C = O keto), 148.2 (C-2), 142.5 (C-4), 140.1 (C-10a), 138.0 (C-9), 132.7 (C-13), 132.1 (2 × C-15), 130.8 (C-8a), 130.6 (2 × C-14), 130.1 (C-7), 130.0 (C-6), 129.4 (C-8), 128.9 (C-16), 127.9 (C-4a), 127.8 (C-4b), 124.2 (C-5), 122.8 (C-3), 116.4 (C-10), 63.8 (CH_2_); Anal. Calcd. for C_21_H_15_Br_2_NO C, 55.17; H, 3.31; N, 3.06. Found C, 55.07; H, 3.36; N, 3.16.

#### 3.2.14. 1-(4-Chlorophenacyl)benzo[f]quinolin-1-ium bromide (**3n**)

Yellowish powder; yield: 79%; m.p. 192–196 °C; IR, ν_max_ 3020, 2931, 1688, 1586, 1341, 1088, 759 cm^−1^; ^1^H-NMR (DMSO-*d*_6_) δ 10.28 (1H, d, *J* = 8.5 Hz, H-4), 9.47 (1H, d, *J* = 5.5 Hz, H-2), 9.18 (1H, d, *J* = 8.5 Hz, H-5), 8.64 (1H, d, *J* = 9.5 Hz, H-9), 8.47 (1H, atd, *J* = 8.5 Hz, *J* = 6.0 Hz, H-3), 8.36 (1H, d, *J* = 9.5 Hz, H-10), 8.31 (1H, d, *J* = 8.0 Hz, H-8), 8.18 (2H, d, *J* = 8.5 Hz, 2 × H-14), 8.05 (1H, t, *J* = 8.0 Hz, *J* = 7.0 Hz, H-6), 7.99 (1H, t, *J* = 8.0 Hz, *J* = 7.0 Hz, H-7), 7.80 (2H, d, *J* = 8.5 Hz, 2 × H-15), 7.08 (2H, s, CH_2_); ^13^C-NMR (DMSO-*d*_6_) δ 189.9 (C = O keto), 148.2 (C-2), 142.6 (C-4), 140.1 (C-10a), 139.6 (C-16), 138.1 (C-9), 132.4 (C-13), 130.8 (C-8a), 130.6 (2 × C-14), 130.1 (C-7, C-6), 129.4 (C-8), 129.2 (2 × C-15), 128.0 (C-4a), 127.9 (C-4b), 124.3 (C-5), 122.8 (C-3), 116.4 (C-10), 63.8 (CH_2_); Anal. Calcd. for C_21_H_15_BrClNO C, 61.11; H, 3.66; N, 3.39. Found C, 61.01; H, 3.60; N, 3.29.

#### 3.2.15. 1-(4-Fluorophenacyl)benzo[f]quinolin-1-ium bromide (**3o**)

Cream-colored powder; yield: 87%; m.p. 143–145 °C; IR, ν_max_ 3048, 2969, 1694, 1593, 1335, 1221, 1152 cm^−1^; ^1^H-NMR (DMSO-*d*_6_) δ 10.27 (1H, d, *J* = 8.5 Hz, H-4), 9.51 (1H, d, *J* = 6.0 Hz, H-2), 9.16 (1H, d, *J* = 8.0 Hz, H-5), 8.63 (1H, d, *J* = 10.0 Hz, H-9), 8.47 (1H, atd, *J* = 8.5 Hz, *J* = 6.5 Hz, H-3), 8.34 (1H, d, *J* = 10.0 Hz, H-10), 8.28 (3H, m, H-8, 2 × H-14), 8.04 (1H, t, *J* = 7.5 Hz, H-6), 7.97 (1H, t, *J* = 7.5 Hz, H-7), 7.56 (2H, t, *J* = 8.5 Hz, *J* = 9.0 Hz, 2 × H-15), 7.11 (2H, s, CH_2_); ^13^C-NMR (DMSO-*d*_6_) δ 189.5 (C = O keto), 165.93 (d, *J* = 101 Hz, C-16), 148.2 (C-2), 142.5 (C-4), 140.1 (C-10a), 138.1 (C-9), 131.9 (d, *J* = 4.0 Hz, 2 × C-14), 130.8 (C-8a), 130.4 (ad, *J* = 2.0 Hz, C-13), 130.1 (C-7, C-6), 129.4 (C-8), 128.0 (C-4a), 127.9 (C-4b), 124.3 (C-5), 122.8 (C-3), 116.4 (C-10), 116.26 (d, *J* = 9.0 Hz, 2 × C-15), 63.8 (CH_2_); Anal. Calcd. for C_21_H_15_BrFNO C, 63.65; H, 3.82; N, 3.53. Found C, 63.75; H, 3.77; N, 3.43.

### 3.3. Antimicrobial Assay

#### 3.3.1. Disk-Diffusion Method

For inoculum preparation, reference microbial cultures of bacteria (*Staphylococcus aureus* ATCC 25923, *Escherichia coli* ATCC 25922) and fungi (*Candida albicans* ATCC 10231) were employed. A number of approximately five colonies from each type of culture were used to inoculate 10 mL of Mueller Hinton (MH) agar (for antibacterial tests) and Sabouraud agar (for antifungal tests). Using a DU 730 spectrophotometer (Beckman Coulter, city, state abbrev if USA, country, λ = 600 nm), the turbidity of the inoculum was adjusted to a 0.5 McFarland standard (1–2 × 10^8^ CFU/mL for bacteria and 1–5 × 10^6^ CFU/mL for *Candida*), and the inoculum was transferred, in a 1 mL volume, onto the surface of the growth media specific for bacteria (MH) and fungi (Sabouraud). Once the inoculum was absorbed, sterile paper disks of approximately 6 mm in diameter and impregnated with 10 µL of antibacterial compound (dissolved in 3% DMSO) were placed on the surface of the culture media; for all the tested compounds, the concentration used was 25 mg/mL. Following incubation at the optimal temperatures for bacteria and fungi, of 37 °C and 28 °C, respectively, for 24 h (bacteria) and 72 h (fungi), the diameters of the inhibition zones were measured using a ruler. The controls were prepared in the same growth conditions (i.e., C+: sterile filter paper disks impregnated with antibiotics inducing sensitivity in the organisms under investigation, namely penicillin 10 IU for *Staphylococcus aureus*, carbenicillin 100 µg/mL for *Escherichia coli* and nystatin 500,000 IU for *Candida albicans*, and C−: sterile filter paper disks with no antimicrobial compounds).

#### 3.3.2. Broth Microdilution Method for Determining the Minimum Inhibitory Concentration (MIC)

The working technique involves the use of a 96-well microtiter plate (microdilution). In each well of the plate, 80 µL of growth medium MH, 10 µL of microbial inoculum (*Staphylococcus aureus* ATCC 25923, *Escherichia coli* ATCC 25922, or *Candida albicans* ATCC 10231) prepared in the same manner as in the diffusion test (i.e., by diluting the standardized microbial suspension adjusted to a 0.5 McFarland standard), and 100 µL of antimicrobial substance to be tested were transferred by pipetting, in different concentrations. For this purpose, double dilutions of the antimicrobial agent were made in the DMSO 3%, starting with the 25 µg/mL dilution (e.g., 12.5 µg/mL, 6.25 µg/mL, 3.12 µg/mL, 1.56 µg/mL, 0.78 µg/mL and so on). For each tested microorganism, a positive control C+ (containing 80 µL of MH growth medium, 10 µL of diluted microbial culture, 100 µL successive double dilutions of antibiotic) and a negative one C− (containing 80 µL of MH growth medium and 10 µL of diluted microbial culture) were prepared. Following the incubation of the microplates at 37 °C for 24 h (for *Staphylococcus aureus* ATCC 25923 and *Escherichia coli* ATCC 25922) and at 28 °C for 72 h (for *Candida albicans* ATCC 10231), 10 µL of resazurin were added in each well. The samples were incubated once again at the optimal temperature for each microorganism for one hour. The colour of the indicator turned from purple to pink. Resazurin is a colorimetric indicator for cell viability widely applied for monitoring cell proliferation. The redox dye, resazurin, enters the cytosol in the oxidized form (purple-blue) and is converted to the reduced form, resorufin (pink).

## 4. Conclusions

We report herein the design, synthesis, experimental and in silico evaluation of the antibacterial and antifungal activity of some new benzo[f]quinoline derivatives. Two classes of benzo[f]quinolinium derivatives (salts **BQS** and cycloadducts **PBQC**) were designed and obtained via a direct and facile two step procedure: quaternization followed by a cycloaddition reaction. The synthesized compounds were characterized by elemental and spectral analysis (FT-IR, ^1^H-NMR, ^13^C-NMR). The antifungal assays revealed that the **BQS** salts have an excellent quasi-nonselective antifungal activity against the fungus *Candida albicans*, some of them higher that the control drug nystatin. The antibacterial assay revealed that the **BQS** salts have a very good antibacterial activity against the Gram positive germ *Staphylococcus aureus* while the activity against the Gram negative germ *Escherichia coli* is negligible. The cycloadducts **PBQC 4** are inactive. Analysis of the biological data reveals some interesting SAR correlations between the structures and their antimicrobial activity. The in silico studies furnished important data concerning the pharmacodynamics, pharmacokinetics and ADMET parameters of the **BQS** salts. Study of the de interaction of each **BQS** salt **3a-o** with ATP synthase in the formed complex, reveal that salts **3j**, **3i,** and **3n** have the best-fit in complex with ATP synthase. Study of the de interaction of each **BQS** salt **3a-o** with TOPO II in the formed complex, revealed that salts **3j** and **3n** have the best-fit in complex with TOPO II. The in silico ADMET studies reveal that the **BQS** salts have excellent drug-like properties, low toxicity profiles, excellent blood—brain barrier permeability, an excellent oral absorption and a good solubility. Overall, the experimental and in silico studies indicated that compounds **3e** and **3f** (from the aliphatic series) and **3i**, **3j** and **3n** (from the aromatic series), are promising leading drug candidates.

## Figures and Tables

**Table 1:** Main spectral data of **BQS** salts **3a**–**o**.

Salt	IR, cm^−1^	H-2	H-4	H-11 (CH_2_)	^13^C-NMR
**3a**	1626 (ν_CO_)	9.47	10.18	5.91	166.0 (C-12: CO), 148.5 (C-2), 142.1 (C-4), 59.3 (C-11: CH_2_)
**3b**	1743 (ν_CO_)	9.62	10.23	6.33	166.6 (C-12: CO), 148.3 (C-2), 142.9 (C-4), 57.9 (C-11: CH_2_)
**3c**	1741 (ν_CO_)	9.60	10.25	6.30	166.1 (C-12: CO), 148.4 (C-2), 142.8 (C-4), 57.9 (C-11: CH_2_)
**3d**	1736 (ν_CO_)	9.39	10.20	6.43	199.7 (C-12: CO), 147.8 (C-2), 144.0 (C-4), 66.0 (C-11: CH_2_)
**3e**	1723 (ν_CO_)	9.40	10.21	6.42	202.3 (C-12: CO), 147.8 (C-2), 142.2 (C-4), 65.3 (C-11: CH_2_)
**3f**	1708 (ν_CO_)	9.46	10.24	6.68	207.0 (C-12: CO), 148.2 (C-2), 142.3 (C-4), 63.0 (C-11: CH_2_)
**3g**	1682 (ν_CO_)	9.45	10.20	7.05	190.6 (C-12: CO), **148**.2 (C-2), 142.5 (C-4), 63.9 (C-11: CH_2_)
**3h**	1677 (ν_CO_)	9.55	10.25	7.12	190.2 (C-12: CO), 148.2 (C-2), 142.4 (C-4), 63.8 (C-11: CH_2_)
**3i**	1673 (ν_CO_)	9.52	10.25	7.08	188.9 (C-12: CO), 148.2 (C-2), 142.4 (C-4), 63.5 (C-11: CH_2_)
**3j**	1689 (ν_CO_)	9.56	10.28	7.18	190.3 (C-12: CO), 148.2 (C-2), 142.5 (C-4), 63.9 (C-11: CH_2_)
**3k**	1706 (ν_CO_)	9.51	10.28	7.15	190.3 (C-12: CO), 148.2 (C-2), 142.6 (C-4), 64.1 (C-11: CH_2_)
**3l**	1717 (ν_CO_)	9.52	10.29	7.18	190.1 (C-12: CO), 148.2 (C-2), 142.7 (C-4), 64.1 (C-11: CH_2_)
**3m**	1696 (ν_CO_)	9.52	10.27	7.11	190.1 (C-12: CO), 148.2 (C-2), 142.5 (C-4), 63.8 (C-11: CH_2_)
**3n**	1698 (ν_CO_)	9.47	10.28	7.08	189.9 (C-12: CO), 148.2 (C-2), 142.6 (C-4), 63.8 (C-11: CH_2_)
**3o**	1694 (ν_CO_)	9.51	10.27	7.11	189.5 (C-12: CO), 148.2 (C-2), 142.5 (C-4), 63.8 (C-11: CH_2_)

**Table 2:** The antibacterial and antifungal activity for **BQS** salts **3a–o** and **PBQC** cycloadducts **4e** and **4g**.

Cpd/R	^a^ Diameter of Inhibition Zone (mm)		
	*S. aureus* ATCC 25923	*E. coli* ATCC 25922	*C. albicans* ATCC 10231
**3a**/-NH_2_	0	0	10.5 ± 0.5
**3b**/-OMe	19.5 ± 1.5	17 ± 1.73	**20 ± 1.5**
**3c**/-OEt	**20 ± 1**	18.5 ± 1.5	**22 ± 1.25**
**3d**/-Me	16 ± 1	11 ± 1	**20 ± 1.80**
**3e**/-Et	17 ± 1.73	12.5 ± 1.5	**30 ± 2**
**3f**/-*t*-Bu	**20 ± 1**	15.5 ± 2	**27.5 ± 1.5**
**3g**/-C_6_H_5_	18 ± 1.5	13 ± 1.5	**20 ± 1.8**
**3h**/-C_6_H_4_(Me)_p_	**20.5 ± 1.5**	17 ± 2	**21.5 ± 1.8**
**3i**/-C_6_H_4_(OMe)_p_	**21.5 ± 1.73**	18 ± 1.73	**22 ± 1**
**3j**/-C_6_H_4_(C_6_H_5_)_p_	15 ± 1.5	13 ± 1.73	17 ± 1
**3k**/-C_6_H_4_(CN)_p_	15 ± 2.5	12.5 ± 1.25	15 ± 1.73
**3l**/-C_6_H_4_(NO_2_)_p_	15 ± 2	14 ± 2.6	17 ± 1
**3m**/-C_6_H_4_(Br)_p_	16 ± 1.5	12 ± 1.5	19 ± 1.5
**3n**/-C_6_H_4_(Cl)_p_	**21 ± 1.5**	18 ± 1.32	**22.5 ± 1**
**3o**/-C_6_H_4_(F)_p_	14 ± 1.5	10.5 ± 1.5	14 ± 0.5
**4e1**	0	0	0
**4e2**	0	0	8 ± 1
**4g1**	0	0	0
**4g2** **C+**	0 44 ± 1	0 46 ± 1.33	0 21 ± 1

All values represented in the table are average of results of five separately conducted experiments. Bold and underline means very active, while underline means active. ^a^ Diameter of inhibition zone (mm); X ± SD, mean of five measurements ± standard deviation. C+: Penicillin 10 IU for *Staphylococcus aureus*, carbenicillin 100 µg/mL for *Escherichia coli* and nystatin 500,000 IU for *Candida albicans*.

**Table 3:** The minimum inhibitory concentration (MIC) for **BQS** salts **3b**–**e**, **3g**–**k**, **n** (µg/mL).

Strain		MIC (µg/mL)									
	C+	3b	3c	3d	3e	3g	3h	3i	3j	3k	3n
*S. aureus ATCC 25923*	0.5	1.56	0.39	1.56	0.78	0.195	0.195	**0.00304**	0.39	1.19	*0.0975*
*E. coli ATCC 25922*	0.25	1.56	0.78	1.56	0.78	0.195	0.195	**0.00152**	0.78	1.39	0.195
*C. albicans ATCC 10231*	1.56	3.12	0.78	3.12	3.12	0.139	0.195	**0.0575**	0.78	0.81	0.195

C+: Penicillin for *Staphylococcus aureus*, Carbenicillin for *Escherichia coli* and Nystatin for *Candida albicans*. Bold and underline means very active, while underline means active.

**Table 4:** ADME properties computed for **BQS 3a**–**o**.

#	*Structures*	*QP* *Logs*	*ClQP* *Logs*	*QP* *Log* *HERG*	*QP* *PCaco*	*QP* *Logbb*	*QP* *PMDCK*	*QP* *Logkp*	*Metab.*	*QP* *Log* *Khsa*	*%Ab-Sorbtion*	*Rule of 5*	*Rule of 3*
1	**3a**	−1.616	−2.741	−3.322	−3.322	−3.322	512.729	512.729	512.729	512.729	512.729	0	0
2	**3b**	−4.078	−3.835	−5.248	−5.248	−5.248	2017.43	2017.43	2017.43	2017.43	2017.43	0	0
3	**3c**	−4.668	−4.133	−5.585	−5.585	−5.585	2333.6	2333.6	2333.6	2333.6	2333.6	0	0
4	**3d**	−3.555	−3.495	−4.966	−4.966	−4.966	2001.37	2001.37	2001.37	2001.37	2001.37	0	0
5	**3e**	−3.805	−3.793	−5.012	−5.012	−5.012	2500.67	2500.67	2500.67	2500.67	2500.67	0	0
6	**3f**	−4.066	−4.091	−4.992	−4.992	−4.992	2271.19	2271.19	2271.19	2271.19	2271.19	0	0
7	**3g**	−4.291	−4.766	−5.876	−5.876	−5.876	1877	1877	1877	1877	1877	0	0
8	**3h**	−2.913	−3.555	−5.857	−5.857	−5.857	176.027	176.027	176.027	176.027	176.027	0	0
9	**3i**	−4.495	−5.463	−5.52	−5.52	−5.52	2244.4	2244.4	2244.4	2244.4	2244.4	0	0
10	**3j**	−6.680	−6.742	−7.42	−7.42	−7.42	2096.68	2096.68	2096.68	2096.68	2096.68	1	1
11	**3k**	−5.748	−6.008	−6.132	−6.132	−6.132	394.919	394.919	394.919	394.919	394.919	0	1
12	**3l**	−4.902	−5.471	−5.39	−5.39	−5.39	1898.89	1898.89	1898.89	1898.89	1898.89	0	0
13	**3m**	−5.181	−6.442	−5.408	−5.408	−5.408	5868.17	5868.17	5868.17	5868.17	5868.17	1	1
14	**3n**	−5.612	−5.905	−5.947	−5.947	−5.947	5702.91	5702.91	5702.91	5702.91	5702.91	1	1
15	**3o**	−5.223	−5.555	−5.898	−5.898	−5.898	4182.77	4182.77	4182.77	4182.77	4182.77	0	0

## Data Availability

Not applicable.

## Supplementary Materials

The following are available online at https://www.mdpi.com/article/10.3390/ph14040335/s1, Figures S1–S3: The antibacterial activity for **BQS** salts **3i** (OL22), **3h** (OL23), and **QBSC** cycloadducts **4e1** (VB10-29), **4g1** (VB10-30), Tables S1–S4: Docking data used in ligand evaluation and QSAR model, Table S5: Molecular descriptors used in QSAR models evaluation, Table S6: Screening results for the best fit hypothesis of BQS3 interaction with ATP synthase and Topoisomerase II, Figures S4–S33: ^1^H-NMR spectra of compounds **3a–o**.
