# Supplementary material for: Benzoquinoline Derivatives: A Straightforward and Efficient Route to Antibacterial and Antifungal Agents
Source: Pharmaceuticals (Basel). 2021 Apr 6;14(4):335. doi: 10.3390/ph14040335 (PMC8067460; doi:10.3390/ph14040335)

Benzoquinoline derivatives: a straightforward and efficient way to antibacterial and antifungal agents

Vasilichia Antoci [1], Liliana Oniciuc [1], Dorina Amariucai-Mantu [1], Costel Moldoveanu [1], Violeta Mangalagiu [2], Andreea Madalina Amarandei [3], Claudiu N. Lungu [4,*], Simona Dunca [3,*], Ionel I. Mangalagiu [1,2] and Gheorghita Zbancioc [1,*]

[1] Alexandru Ioan Cuza University of Iasi, Faculty of Chemistry, 11 Carol 1st Bvd, Iasi -700506, Romania; vasilichia.antoci@uaic.ro (V.A.); lili_oniciuc@yahoo.com (L.O); dorina.mantu@uaic.ro (D.A.-M.); costel.moldoveanu@uaic.ro (C.M.); ionelm@uaic.ro (I.I.M.); gheorghita.zbancioc@uaic.ro (G.Z.)

[2] Alexandru Ioan Cuza University of Iasi, Institute of Interdisciplinary Research- CERNESIM Centre, 11 Carol I, Iasi, 700506, Romania; ionelm@uaic.ro (I.I.M.) ; violeta.mangalagiu@uaic.ro (V.M.)

[3] Alexandru Ioan Cuza University of Iasi, Faculty of Biology, 11 Carol 1st Bvd, Iasi -700506, Romania; sdunca@uaic.ro (S.D.); aamarandei16@yahoo.com (A.M.A.)

[4] Department of Surgery, Emergency Clinical Hospital, Braila-810325, Romania; lunguclaudiu5555@gmail.com (C.N.L.)

* Correspondence: gheorghita.zbancioc@uaic.ro (G.Z.); lunguclaudiu5555@gmail.com (C.N.L.); sdunca@uaic.ro (S.D.)

Content

1. Figure 1-3.

2. Docking data **-Table S1, S2, S3, S4**

3. Molecular descriptors **-Table S5**

4. Screening results **- Table S6**

5. ^{1}H and ^{13}C NMR spectra **- Figure 4-33**

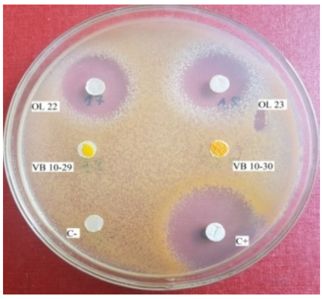

Figure S1. The antibacterial activity for **BQS** salts **3i** (OL22), **3h** (OL23), and **QBSC** cycloadducts **4e1** (VB10-29), **4g1** (VB10-30) against *S. aureus* (C+: positive control; C-: negative control)

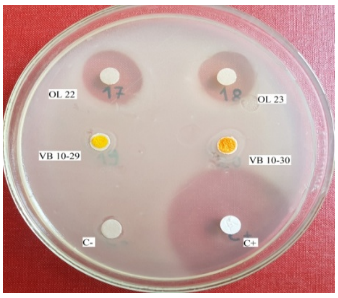

Figure S2. The antibacterial activity for **BQS** salts **3i** (OL22), **3h** (OL23), and **QBSC** cycloadducts **4e1** (VB10-29), **4g1** (VB10-30) against *E. coli* (C+: positive control; C-: negative control)

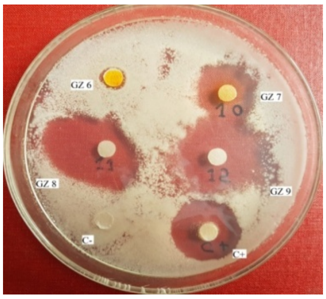

Figure S3. The antifungal activity for **BQS** salts **3e** (GZ8), **3f** (GZ9), **3d** (GZ7) and **QBSC** cycloadduct **4e2** (GZ6) against *C. albicans* (C+: positive control; C-: negative control)

Table S1 Docking data used in ligand evaluation and QSAR model (ATP)

Nr	Compound	Csp2	Csp3	DOF	E-Inter (protein - ligand)	E-Inter total	E-Intra (steric)	E-Intra (tors)	E-Intra (tors, ligand atoms)	E-Intra (vdw)	E-Total
1	3a	13	1	2	-99.811	-99.811	11.7614	1.12776	12.8892	75.5267	-86.9218
2	3b	13	2	3	-108.608	-108.608	14.6733	0.540959	15.2142	101.465	-93.3941
3	3c	13	3	4	-108.899	-108.899	17.257	0.923764	18.1808	102.344	-90.7185
4	3e	13	3	3	-104.79	-104.79	9.94165	0.943848	10.8855	74.9959	-93.9045
5	3d	13	2	2	-99.1188	-99.1188	15.362	0.176939	15.539	80.1552	-83.5798
6	3f	13	4	4	-93.108	-93.108	11.2591	3.23422	14.4933	78.9895	-78.6147
7	3g	19	1	3	-117.324	-117.324	11.0048	1.96883	12.9736	105.62	-104.351
8	3h	19	2	3	-112.931	-112.931	15.2109	3.48031	18.6912	103.198	-94.2394
9	3i	19	2	4	-119.47	-119.47	14.955	2.66604	17.621	110.593	-101.849
10	3j	25	1	4	-140.599	-140.599	15.0258	2.40254	17.4283	88.4046	-123.171
11	3k	20	1	4	-123.642	-123.642	20.19	0.915809	21.1058	113.156	-102.536
12	3l	19	1	4	-117.762	-117.762	5.96118	5.28474	11.2459	79.4192	-106.516
13	3m	19	1	3	-104.149	-104.149	7.85949	2.29569	10.1552	102.669	-93.9938
14	3n	19	1	3	-123.851	-123.851	14.4201	1.9458	16.3659	107.45	-107.485
15	3o	19	1	3	-123.114	-123.114	13.6298	2.24368	15.8735	102.387	-107.241

Table S2 Docking data used in ligand evaluation and QSAR model (ATP)

Nr	Compounds	HBond	HeavyAtoms	N	UnHBond90	OS	PoseEnergy	Steric	Torsions	VdW	Carbonyl	Halogen
1	3a	-2.50991	18	2	-4.91937	0	-88.8302	-97.3011	2	-34.0245	1	0
2	3b	-2.0519	19	1	-5.25518	1	-95.0877	-106.556	3	-37.4008	1	0
3	3c	-1.92612	20	1	-3.54137	1	-91.2257	-106.973	4	-37.9083	1	0
4	3e	0	19	1	-4.43984	0	-96.946	-104.79	3	-33.2238	1	0
5	3d	0	18	1	-2.32137	0	-84.9599	-99.1188	2	-37.0065	1	0
6	3f	-0.182561	20	1	-2.5	0	-80.0558	-92.9254	4	-28.6167	1	0
7	3g	-3.08269	23	1	-3.27895	0	-102.98	-114.242	3	-32.3651	1	0
8	3h	-4.33257	24	1	-5	0	-93.3915	-108.598	3	-36.2744	1	0
9	3i	-2.4289	25	1	-6.67671	1	-105.005	-117.041	4	-39.806	1	0
10	3j	-0.184121	29	1	-0.209049	0	-121.269	-140.415	4	-48.6582	1	0
11	3k	-1.79492	25	2	-6.57073	0	-105.074	-121.847	4	17.4462	1	0
12	3l	-3.24099	26	2	-12.5667	2	-114.961	-114.521	4	-38.3864	1	0
13	3m	0	24	1	0	0	-93.1539	-104.149	3	-33.7435	1	1
14	3n	-0.850418	24	1	-4.02696	0	-109.15	-123.001	3	-37.63	1	1
15	3o	0	24	1	0	0	-105.457	-123.114	3	-39.2591	1	1

Table S3 Docking data used in ligand evaluation and QSAR model (TOPO II)

Nr	Compound	Csp2	Csp3	DOF	E-Inter (protein - ligand)	E-Inter total	E-Intra (steric)	E-Intra (tors)	E-Intra (tors, ligand atoms)	E-Intra (vdw)	E-Total
1	3a	13	1	2	-91.7722	-91.7722	13.029	1.8166	14.8456	75.0644	-76.9266
2	3b	13	2	3	-90.9885	-90.9885	12.8185	1.67737	14.4958	73.244	-76.4927
3	3c	13	3	4	-96.1635	-96.1635	13.6852	2.2905	15.9757	81.139	-80.1878
4	3e	13	3	3	-95.8351	-95.8351	12.0185	0.968386	12.9869	73.6774	-82.8482
5	3d	13	2	2	-110.705	-110.705	13.8016	1.61246	15.414	75.9016	-95.2912
6	3f	13	4	4	-86.0412	-86.0412	5.39488	1.96065	7.35553	79.8012	-78.6857
7	3g	19	1	3	-103.465	-103.465	16.4004	1.29002	17.6904	105.555	-85.7747
8	3h	19	2	3	-122.678	-122.678	16.2592	1.35424	17.6135	107.886	-105.064
9	3i	19	2	4	-105.596	-105.596	15.0423	1.74246	16.7848	108.634	-88.8115
10	3j	25	1	4	-121.469	-121.469	11.1323	2.95651	14.0888	84.9934	-107.381
11	3k	20	1	4	-96.8053	-96.8053	10.1757	1.33882	11.5145	114.176	-85.2908
12	3l	19	1	4	-108.907	-108.907	12.8295	3.18696	16.0165	63.0758	-92.8906
13	3m	19	1	3	-104.453	-104.453	14.6034	1.65082	16.2542	106.53	-88.1991
14	3n	19	1	3	-117.059	-117.059	16.2479	1.55977	17.8077	106.257	-99.2509
15	3o	19	1	3	-116.338	-116.338	13.2986	2.01138	15.31	100.504	-101.028

Table S4 Docking data used in ligand evaluation and QSAR model (TOPO II)

Nr	Compounds	HBond	Heavy Atoms	N	UnHBond 90	OS	PoseEnergy	Steric	Torsions	VdW	Carbonyl	Halogen
**1**	**3a**	0	18	2	0	0	-75.5837	-91.7722	2	-29.2598	1	0
**2**	**3b**	-0.245133	19	1	-2.27677	1	-77.5961	-90.7434	3	-18.956	1	0
**3**	**3c**	0	20	1	-1.355	1	-80.573	-96.1635	4	57.0041	1	0
**4**	**3e**	-0.146578	18	1	-2.78886	0	-84.6571	-95.6886	2	-26.7744	1	0
**5**	**3d**	0	19	1	-2.5	0	-96.0207	-110.705	3	-34.4257	1	0
**6**	**3f**	0	20	1	-5	0	-83.0353	-86.0412	4	-21.4965	1	0
**7**	**3g**	0	23	1	0	0	-84.8832	-103.465	3	-32.4849	1	0
**8**	**3h**	0	24	1	0	0	-103.958	-122.678	3	-37.0318	1	0
**9**	**3i**	0	25	1	0	1	-87.6145	-105.596	4	-31.9538	1	0
**10**	**3j**	-0.817853	29	1	-5	0	-110.142	-120.652	4	-37.3135	1	0
**11**	**3k**	-0.130637	25	2	-5.25879	0	-89.0034	-96.6747	4	-30.3105	1	0
**12**	**3l**	-0.887336	26	2	-7.5	2	-98.1365	-108.02	4	-32.4471	1	0
**13**	**3m**	-1.55611	24	1	-4.6468	0	-89.6431	-102.897	3	-35.1044	1	1
**14**	**3n**	0	24	1	0	0	-96.978	-117.059	3	-33.5685	1	1
**15**	**3o**	0	24	1	-2.5	0	-102.093	-116.338	3	-38.0453	1	1

Table S5 Molecular descriptors used in QSAR models evaluation

Nr.	Descriptor	Explination
1.	mutagenic	Indicates the presence of potentially toxic groups. A non-zero value indicates that the molecule contains a mutagenic group.
2	opr_nring	Number of ring bounds
3	lip_don	Number of NH and OH atoms
4	nmol	Number of conneted components
5	opr_nrot	Number of rotable bound
6	lip_violation	Number of Lipinski's Rule violation
7	opr_leadlike	Drug like properties 1 if<2 , otherwise 0
8	lip_acc	Number of O and N atoms
9	lip_druglike	One if violations<2, if not =0
10	opr_brigd	The nimber of rigid bounds
11	BCUT_PEOE_0	The BCUT descriptors are calculated from the eigenvalues of a modified adjacency matrix. Each ij entry of the adjacency matrix takes the value 1/sqrt(bij) where bij is the formal bond order between bonded atoms i and j. The diagonal takes the value of the PEOE partial charges.
12	GCUT_PEOE_PC+	The GCUT descriptors are calculated from the eigenvalues of a modified graph distance adjacency matrix + wighted by partial charges
13	GCUT_PEOE_0	The GCUT descriptors are calculated from the eigenvalues of a modified graph distance adjacency matrix
14	apol	Sum of the atomic polarizabilities
15	bpol	Sum of the absolute value of the difference between atomic polarizabilities of all bonded atoms in the molecule
16	SlogP	Log of the octanol/water partition coefficient. This property is an atomic contribution model
17	h_logP	Log of the octanol/water partition coefficient using an 8 parameter model based on Hueckel Theory
18	h_logS	Log of the aqueous solubility (mol/L) using a 7 parameter model based on Hueckel Theory
19	h_log_pbo	Sum of log (1 + pi bond order) for all bonds.
20	logS	Log of the aqueous solubility
21	vdw_area	Area of van der Waals surface (Å2) calculated using a connection table approximation.
22	vdw_vol	van der Waals volume (Å3) calculated using a connection table approximation.
23	glob	A value of 1 indicates a perfect sphere while a value of 0 indicates a two- or one-dimensional object.
24	rgyr	Radius of gyration
25	vol	van der Waals volume calculated using a grid approximation
26	VSA	van der Waals surface area
27	vsurf_A	Amphiphilic moment

28	vsurf_CP	Critical packing parameter
29	rsynth	A value in [0,1] indicating the synthetic reasonableness, or feasibility, of the chemical structure. A value of 0 means it is unlikely that the molecule can be synthesized while a value of 1 means that it is likely that the molecule can be synthesized.
30	density	Mass density: molecular weight divided by van der Waals volume as calculated in the vol descriptor.
31	h_mr	Molar refractivity using a 4 parameter model based on Hueckel Theory
32	mr	Molecular refractivity (including implicit hydrogens)
33	diameter	Diameter (biggest dimension) of molecule in Å
34	QP logS	Predicted aqueous solubility, log S. S in mol dm–3 is the concentration of the solute in a saturated solution that is in equilibrium with the crystalline solid.(-6,5-0.5)
35	ClQP logS	Conformation-independent predicted aqueous solubility, log S. S in mol dm–3 is the concentration of the solute in a saturated solution that is in equilibrium with the crystalline solid. .(-6,5-0.5)
36	QP Log HERG	Predicted IC50 value for blockage of HERG K+ channels.(concern below –5)
37	QP PCaco	Predicted apparent Caco-2 cell permeability in nm/sec. Caco-2 cells are a model for the gutblood barrier. QikProp predictions are for non-active transport.(<25 poor, >500 great)
38	QP logBB	Predicted brain/blood partition coefficient. Note: QikProp predictions are for orally delivered drugs so, for example, dopamine and serotonin are CNS negative because they are too polar to cross the blood-brain barrier(-3.0-1.2)
39	QP PMDCK	Predicted apparent MDCK cell permeability in nm/sec. MDCK cells are considered to be a good mimic for the blood-brain barrier. QikProp predictions are for non-active transport. (<25 poor, >500 great)
40	QP logKP	Predicted skin permeability, log Kp(-8.0—1.0)
41	#metab	Number of likely metabolic reactions.(1-8)
42	QP logK hsa	Prediction of binding to human serum albumin.(-1.5-1.5)
43	%Ab sorbtion	Predicted human oral absorption on 0 to 100% scale.The prediction is based on a quantitative multiple linear regression model.(>80% high, <25% poor))
44	Rule of 5	Number of violations of Lipinski's rule of five. The rules are: mol_MW < 500, QPlogPo/w < 5, donorHB ≤ 5, accptHB ≤ 10. Compunds that satisfy these rules are considered drug-like. (The "five" refers to the limits, which are multiples of 5 (max 4)
45	Rule of 3	Number of violations of Jorgensen's rule of three. The three rules are: , QPlogS > -5.7, QP PCaco > 22 nm/s, # Primary Metabolites < 7. Compounds with fewer (and preferably no)

		violations of these rules are more likely to be orally available (max 3)

Table S6 Screening results for the best fit hypothesis of BQS3 interaction with ATP synthase and Topoisomerase II

Virtual screening results for BQS3 ATP hypothesis	Virtual screening results for BQS3 TOPO II hypothesis
virtual screening results for BQS3 ATP	virtual screening results for BQS3 TOI

Figure S4. ^{1}H-NMR spectrum of 1-(2-amino-2-oxoethyl)benzo[f]quinolin-1-ium iodide (**3a**)

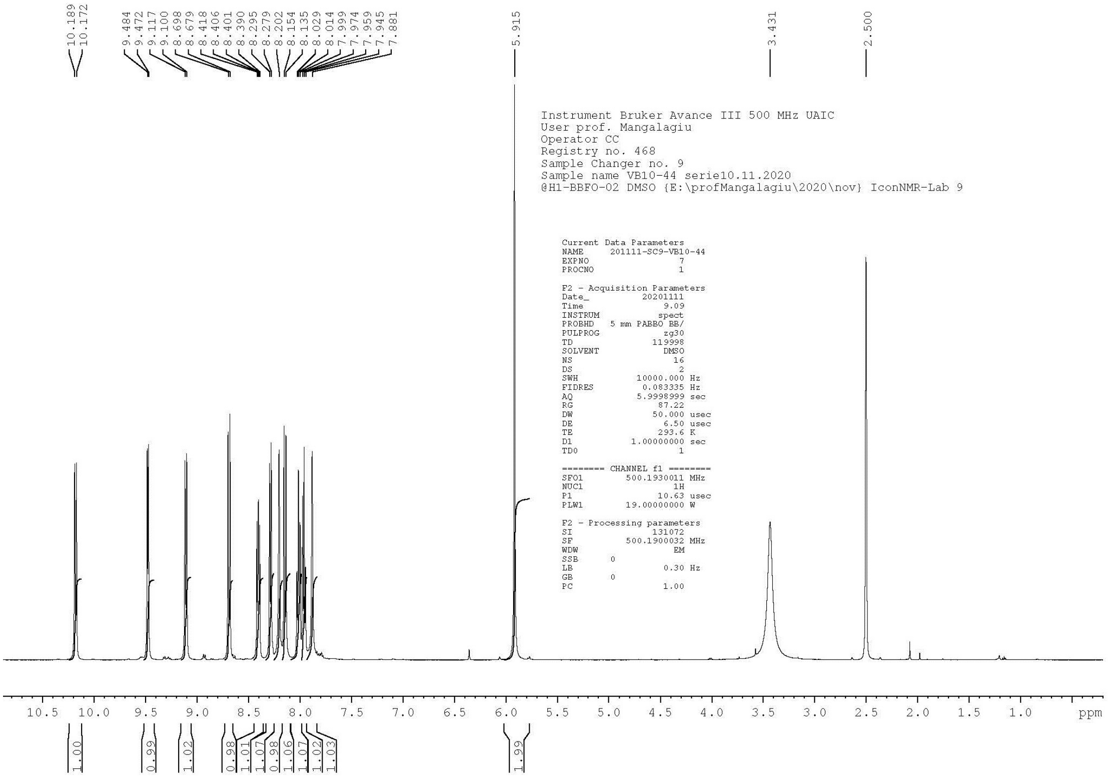

Figure S5. ^{13}C-NMR spectrum of 1-(2-amino-2-oxoethyl)benzo[f]quinolin-1-ium iodide (**3a**)

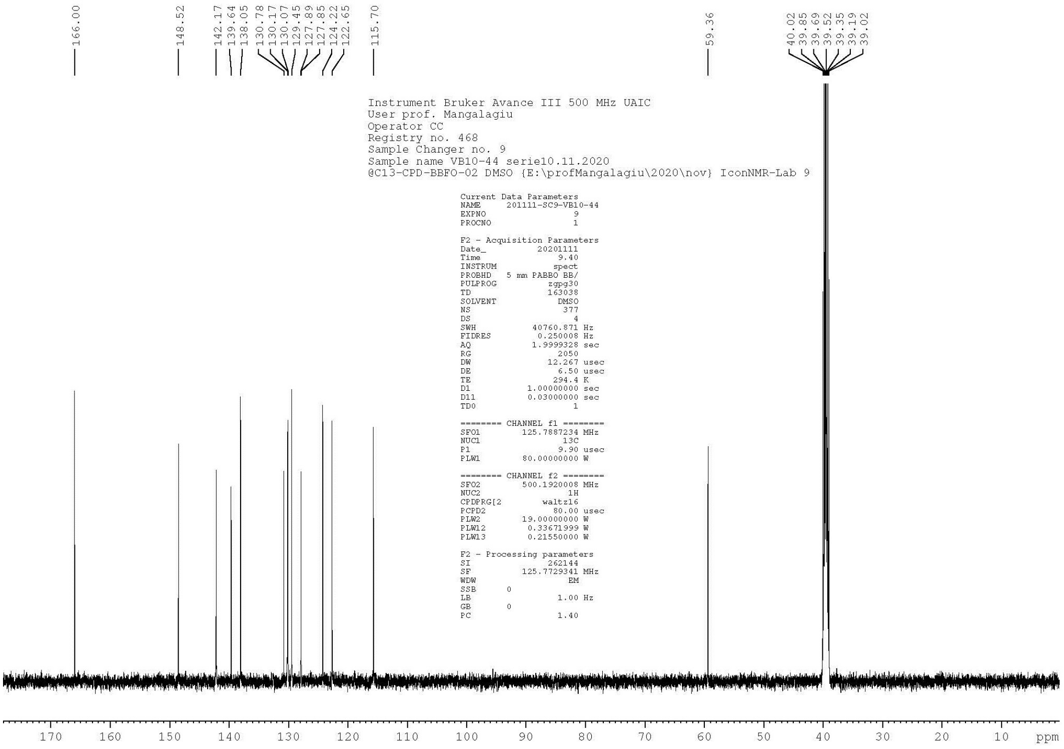

Figure S6. ^{1}H-NMR spectrum of 1-(2-methoxy-2-oxoethyl)benzo[f]quinolin-1-ium (**3b**)

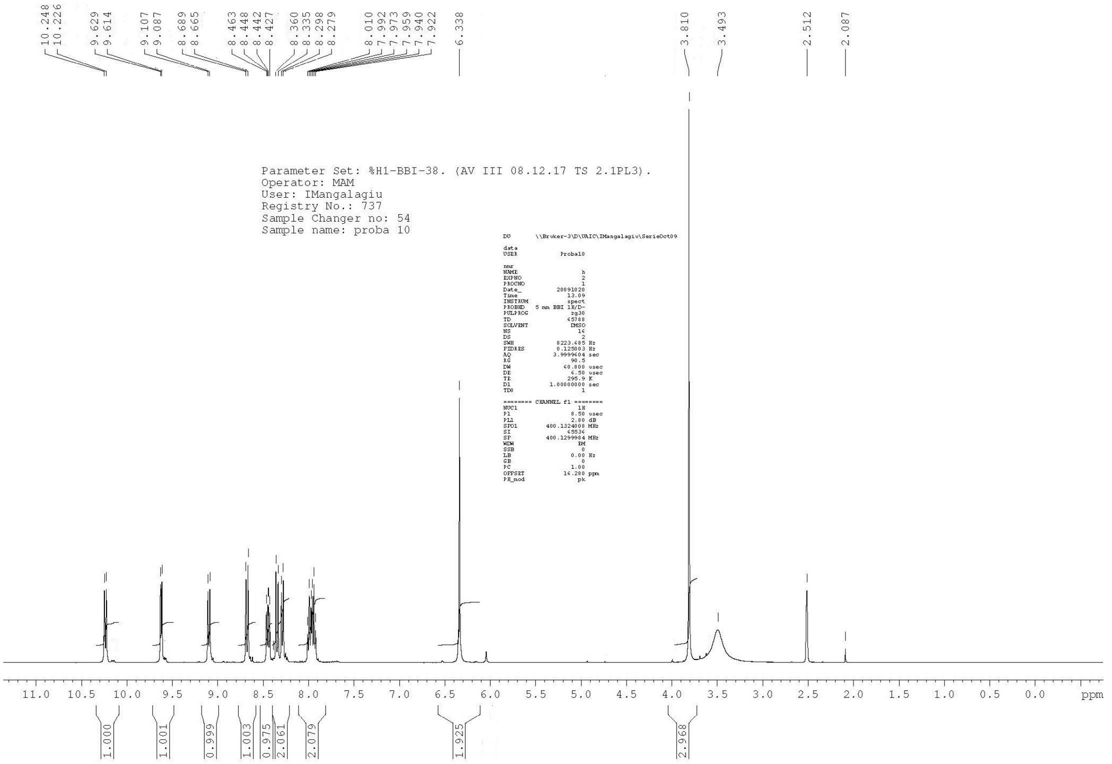

Figure S7. ^{13}C-NMR spectrum of 1-(2-methoxy-2-oxoethyl)benzo[f]quinolin-1-ium (**3b**)

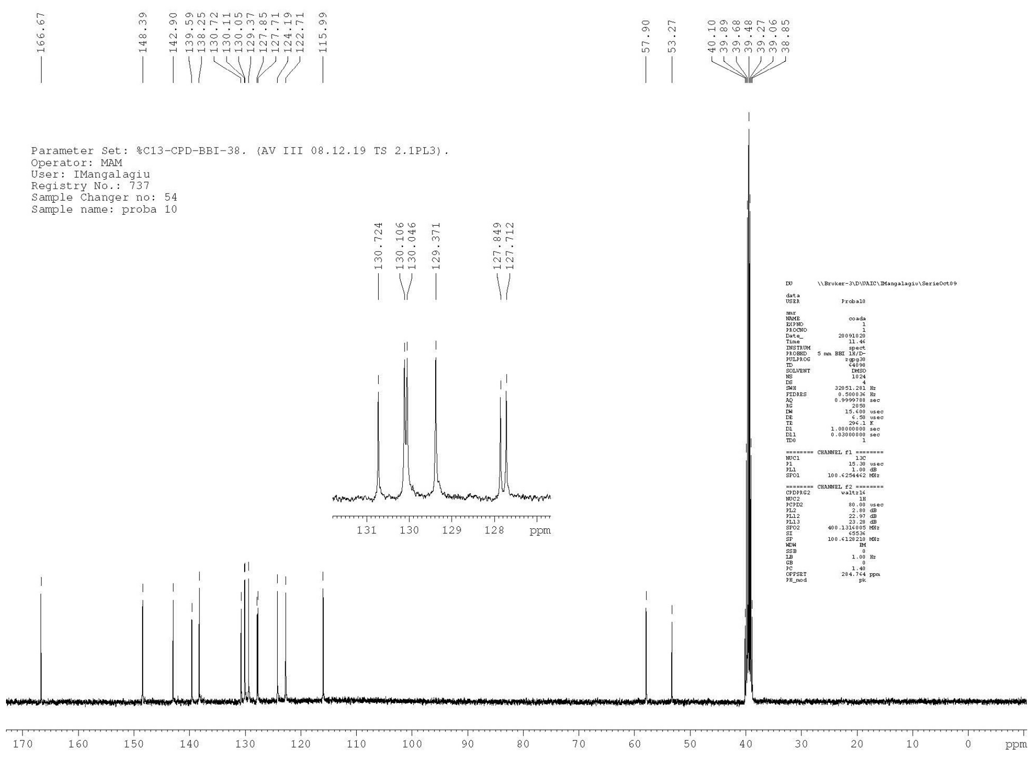

Figure S8. ^{1}H-NMR spectrum of 1-(2-ethoxy-2-oxoethyl)benzo[f]quinolin-1-ium (**3c**)

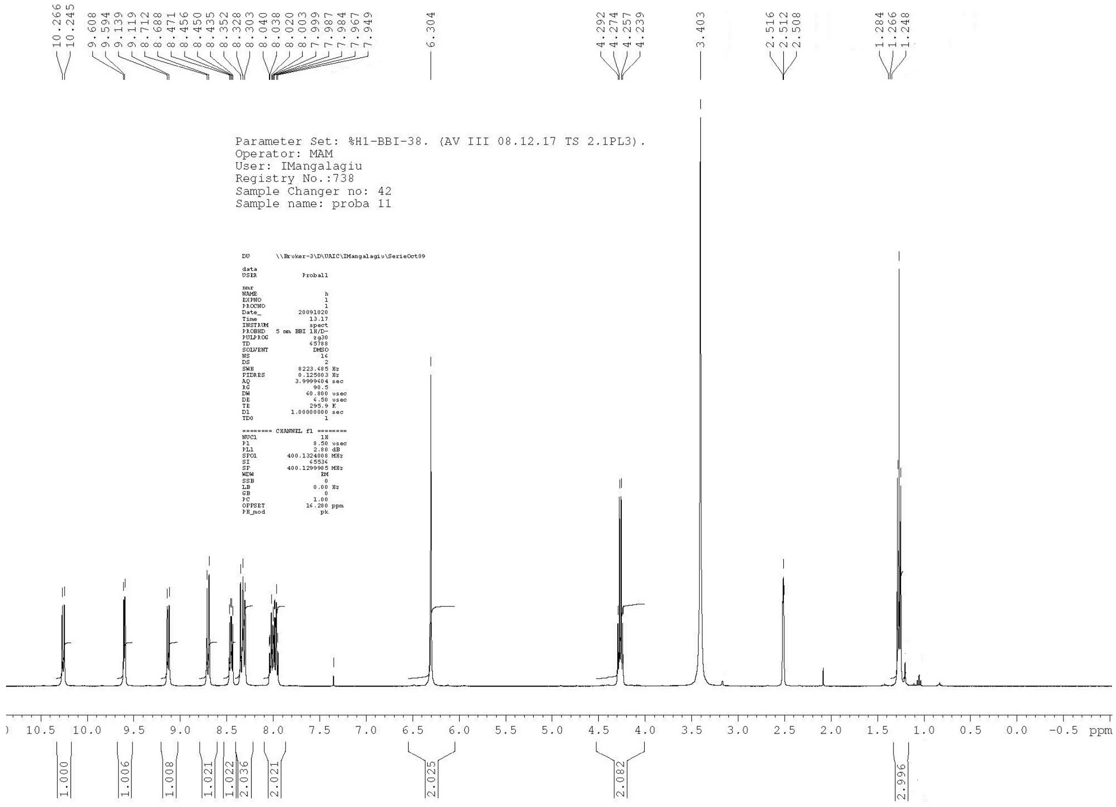

Figure S9. ^{13}C-NMR spectrum of 1-(2-ethoxy-2-oxoethyl)benzo[f]quinolin-1-ium (**3c**)

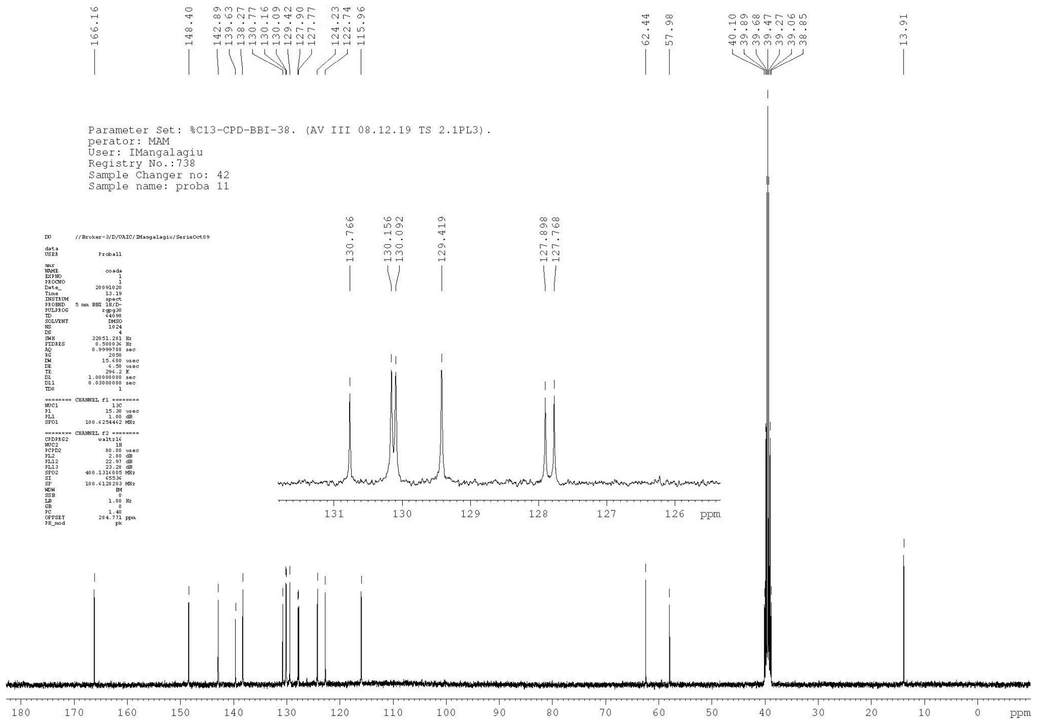

Figure S10. ^{1}H-NMR spectrum of 1-(2-oxopropyl)benzo[f]quinolin-1-ium bromide (**3d**)

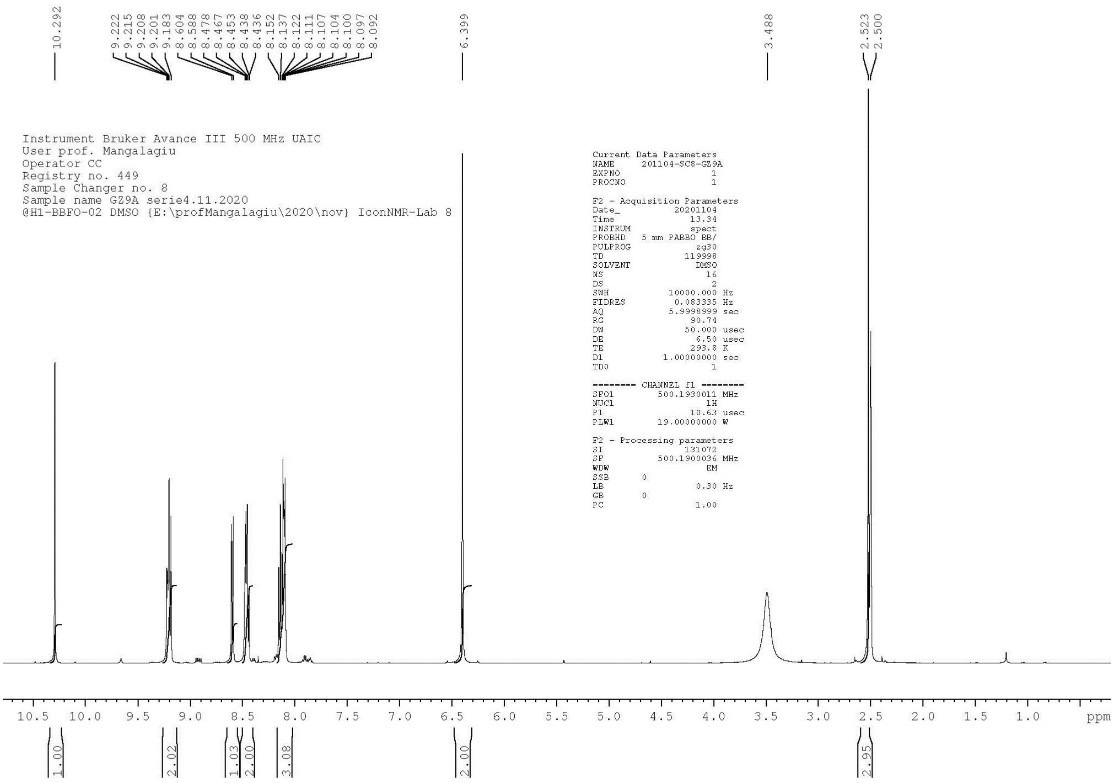

Figure S11. ^{13}C-NMR spectrum of 1-(2-oxopropyl)benzo[f]quinolin-1-ium bromide (**3d**)

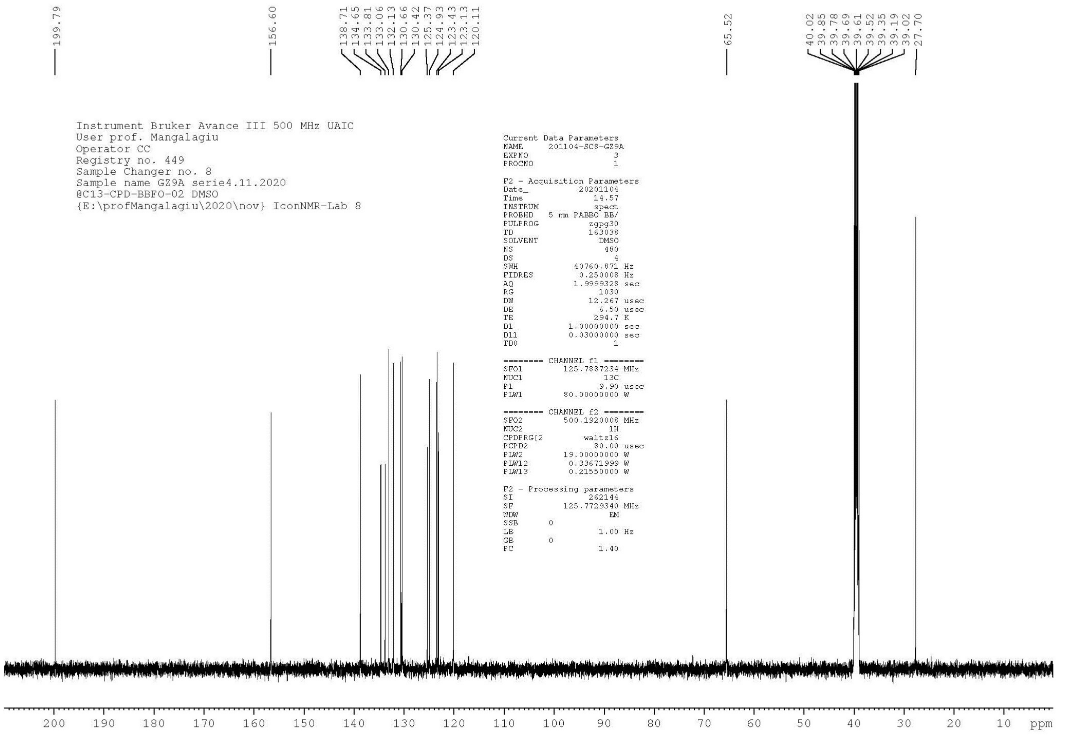

Figure S12. ^{1}H-NMR spectrum of 1-(2-oxobutyl)benzo[f]quinolin-1-ium bromide (**3e**)

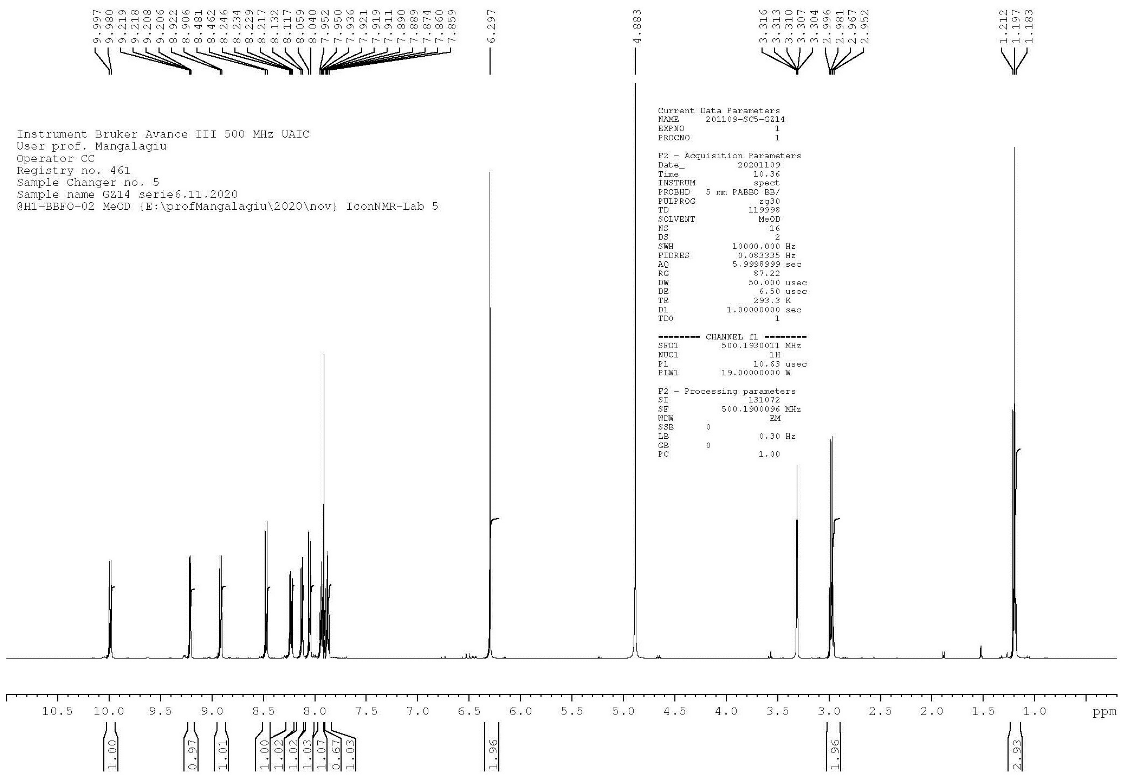

Figure S13. ^{13}C-NMR spectrum of 1-(2-oxobutyl)benzo[f]quinolin-1-ium bromide (**3e**)

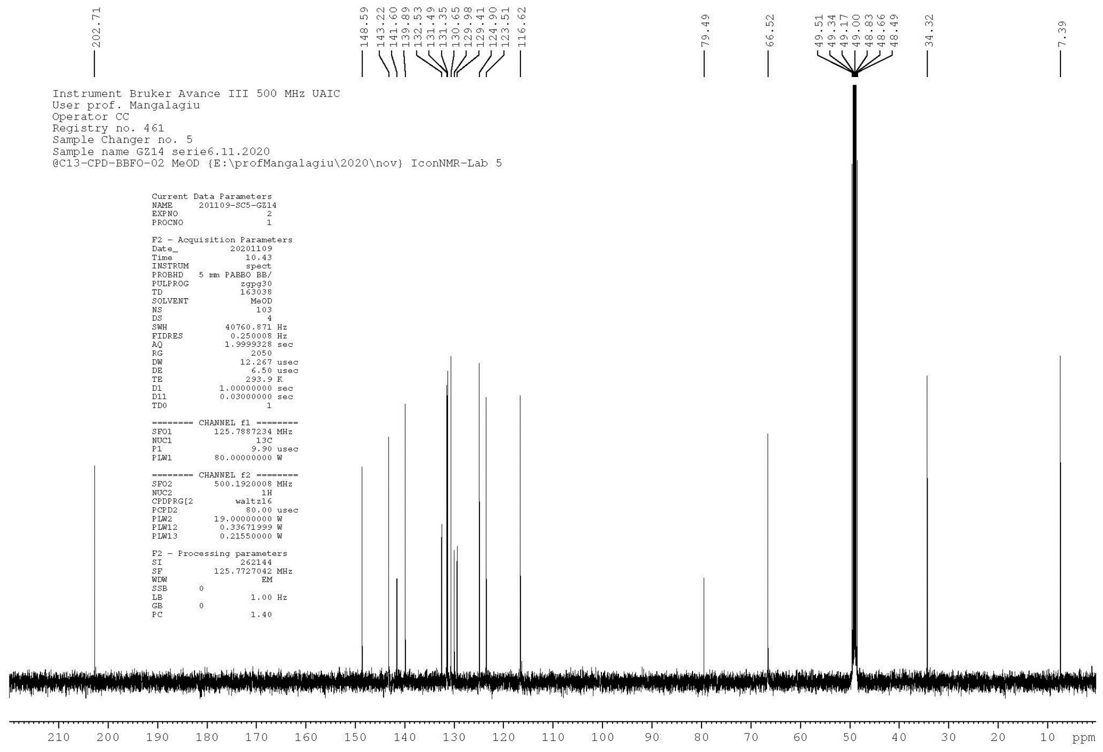

Figure S14. ^{1}H-NMR spectrum of 1-(3,3-dimethyl-2-oxobutyl)benzo[f]quinolin-1-ium bromide **(3f)**

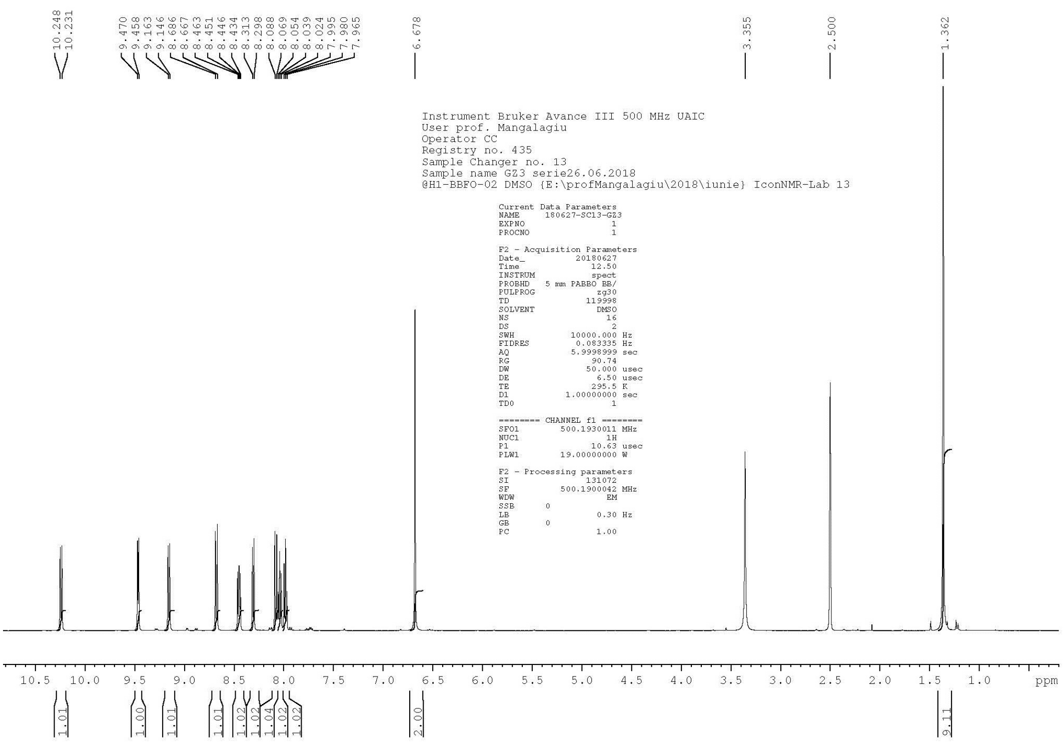

Figure S15. ^{13}C-NMR spectrum of 1-(3,3-dimethyl-2-oxobutyl)benzo[f]quinolin-1-ium bromide **(3f)**

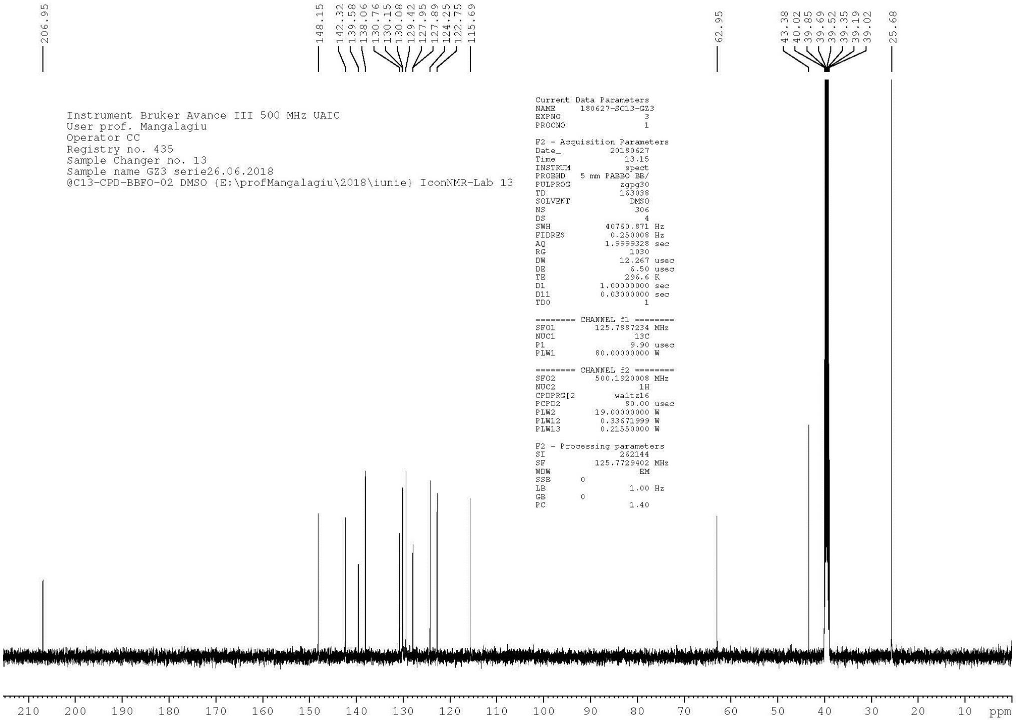

Figure S16. ^{1}H-NMR spectrum of 1-(phenacyl)benzo[f]quinolin-1-ium bromide (**3g**)

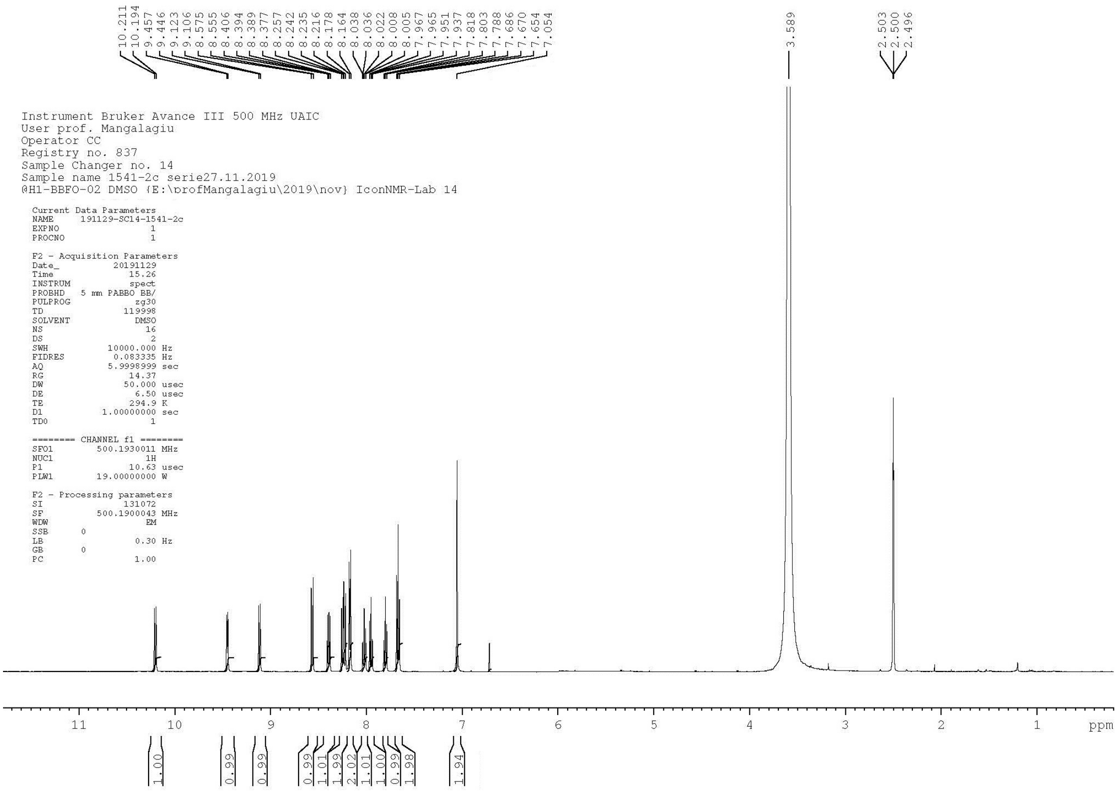

Figure S17. ^{13}C-NMR spectrum of 1-(phenacyl)benzo[f]quinolin-1-ium bromide (**3g**)

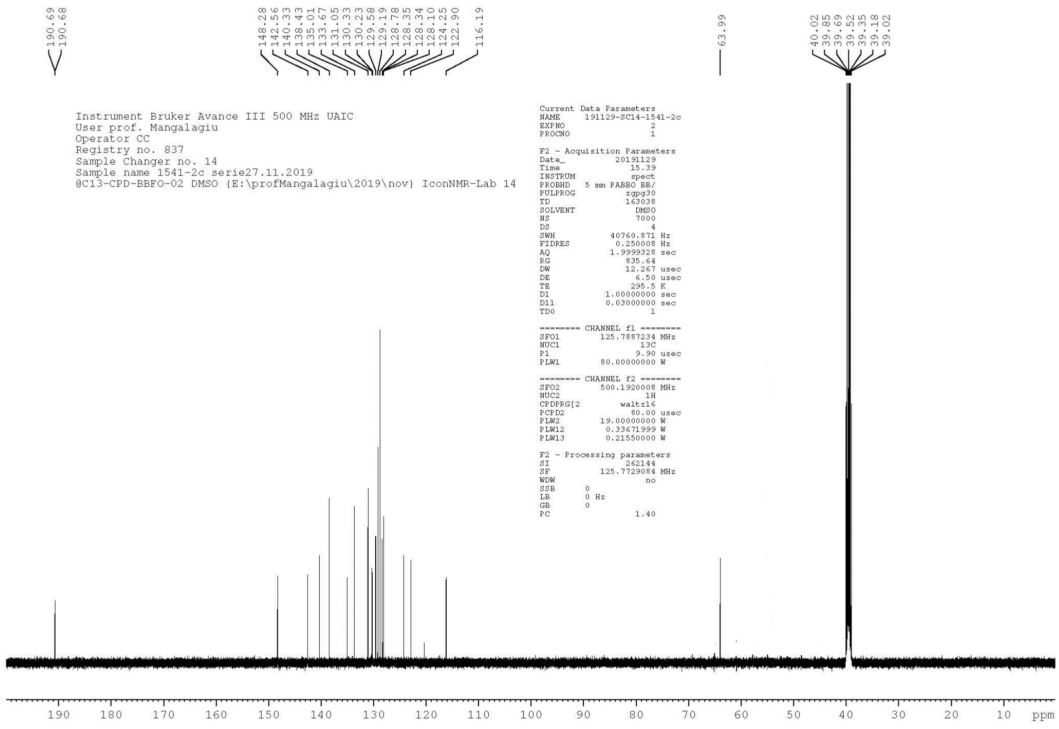

Figure S18. ^{1}H-NMR spectrum of 1-(4-methylphenacyl)benzo[f]quinolin-1-ium bromide (**3h**)

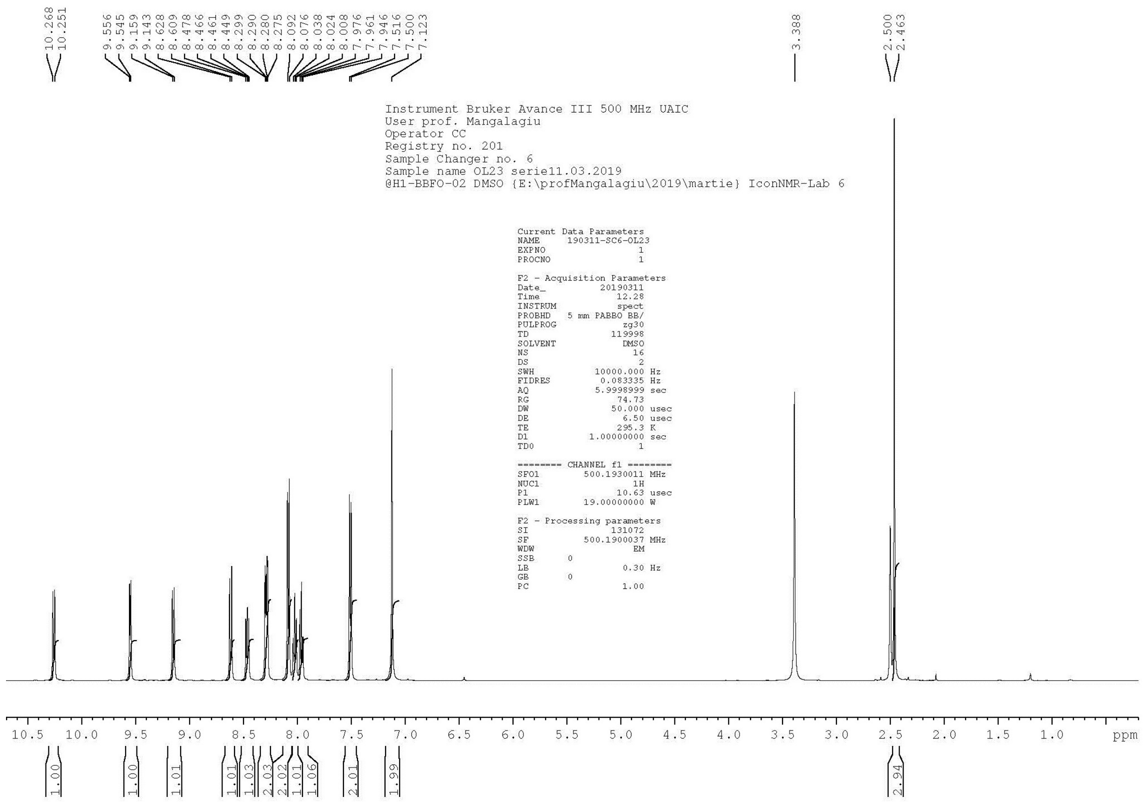

Figure S19. ^{13}C-NMR spectrum of 1-(4-methylphenacyl)benzo[f]quinolin-1-ium bromide (**3h**)

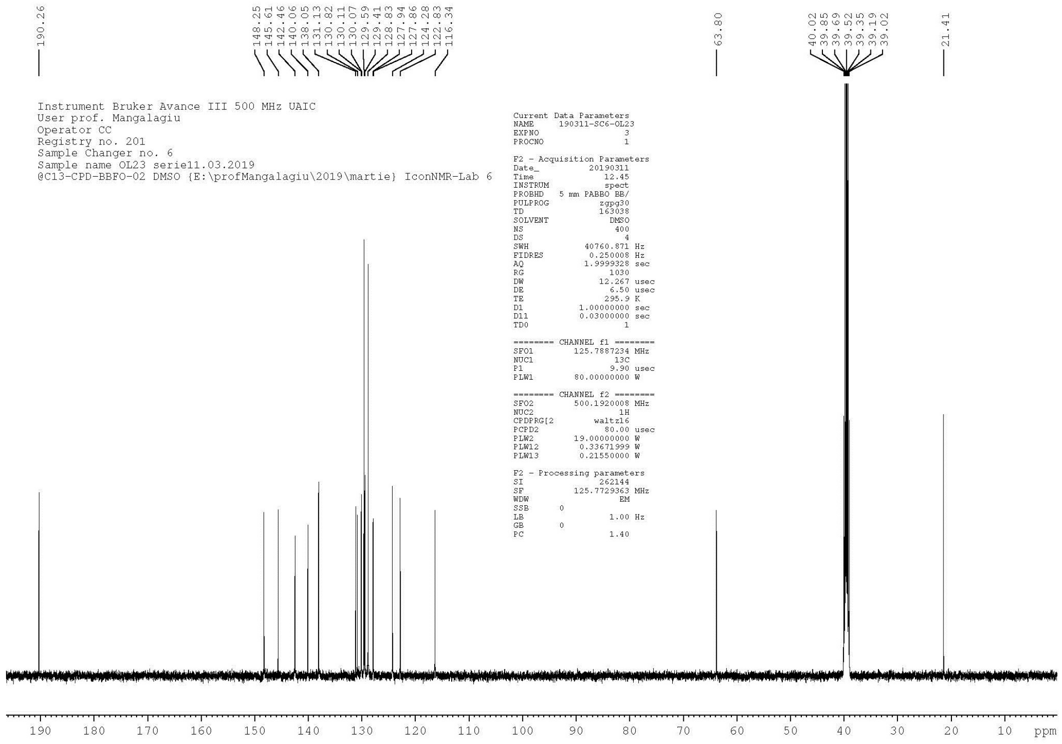

Figure S20. ^{1}H-NMR spectrum of 1-(4-methoxyphenacyl)benzo[f]quinolin-1-ium bromide (**3i**)

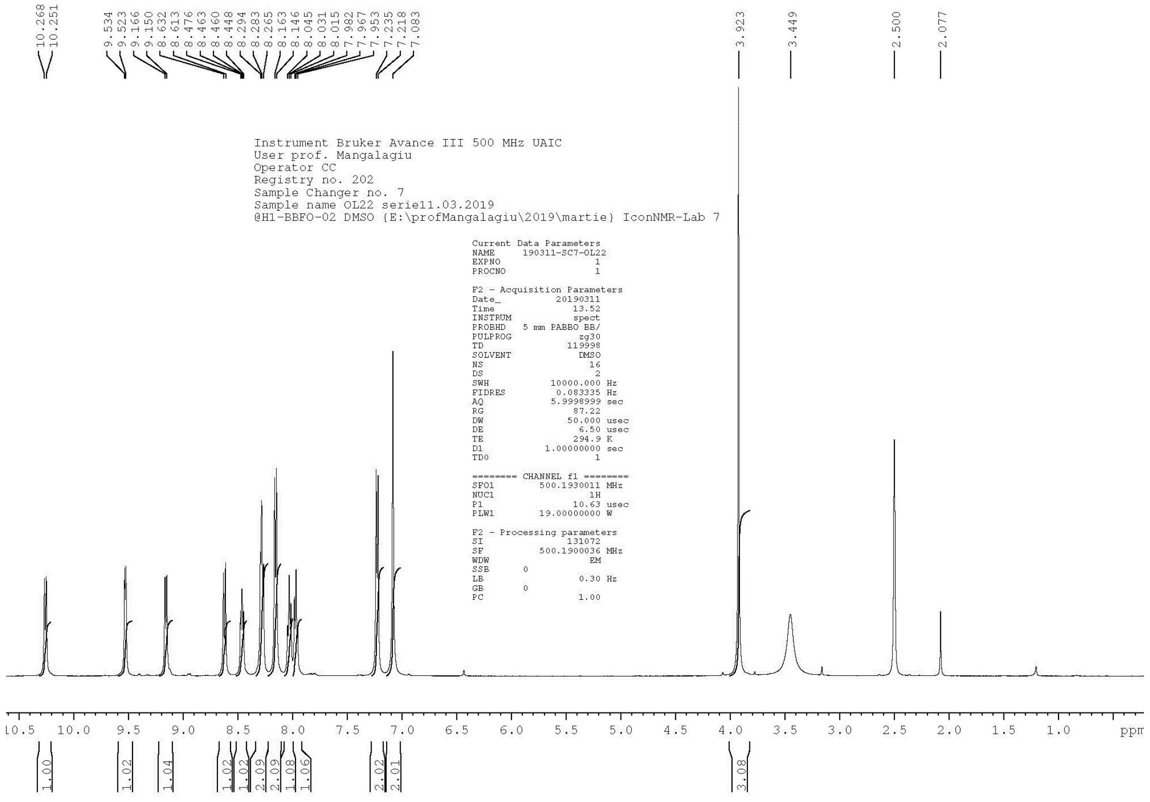

Figure S21. ^{13}C-NMR spectrum of 1-(4-methoxyphenacyl)benzo[f]quinolin-1-ium bromide (**3i**)

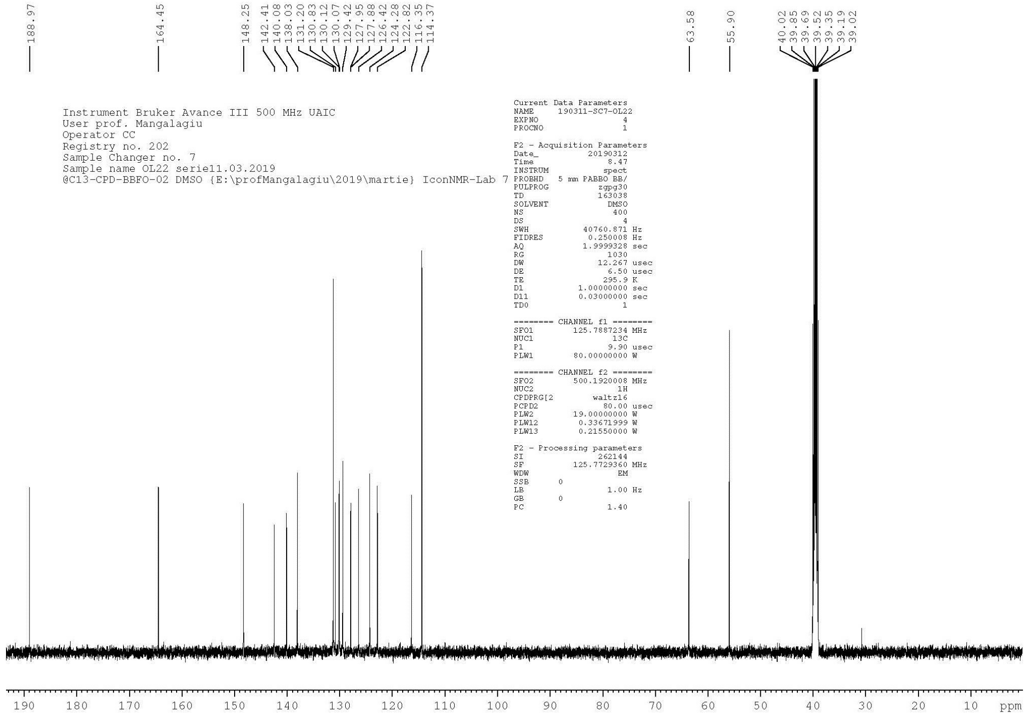

Figure S22. ^{1}H-NMR spectrum of 1-(4-phenylphenacyl)benzo[f]quinolin-1-ium bromide (**3j**)

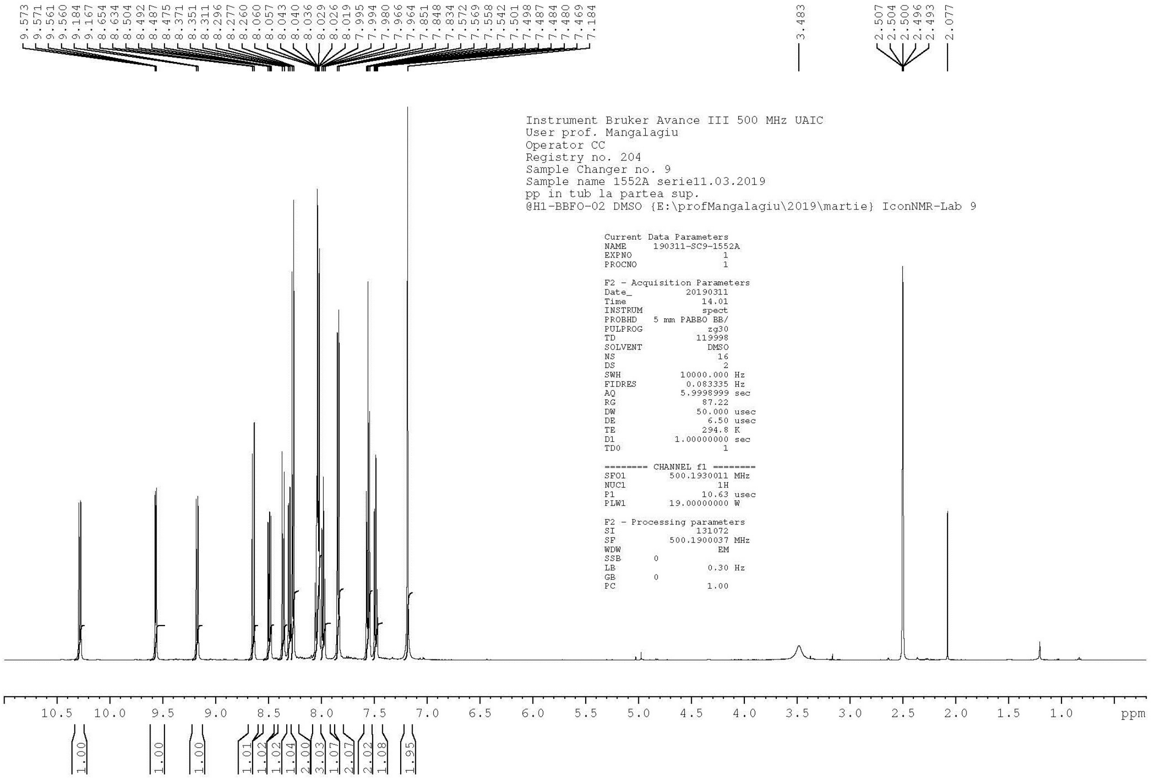

Figure S23. ^{13}C-NMR spectrum of 1-(4-phenylphenacyl)benzo[f]quinolin-1-ium bromide (**3j**)

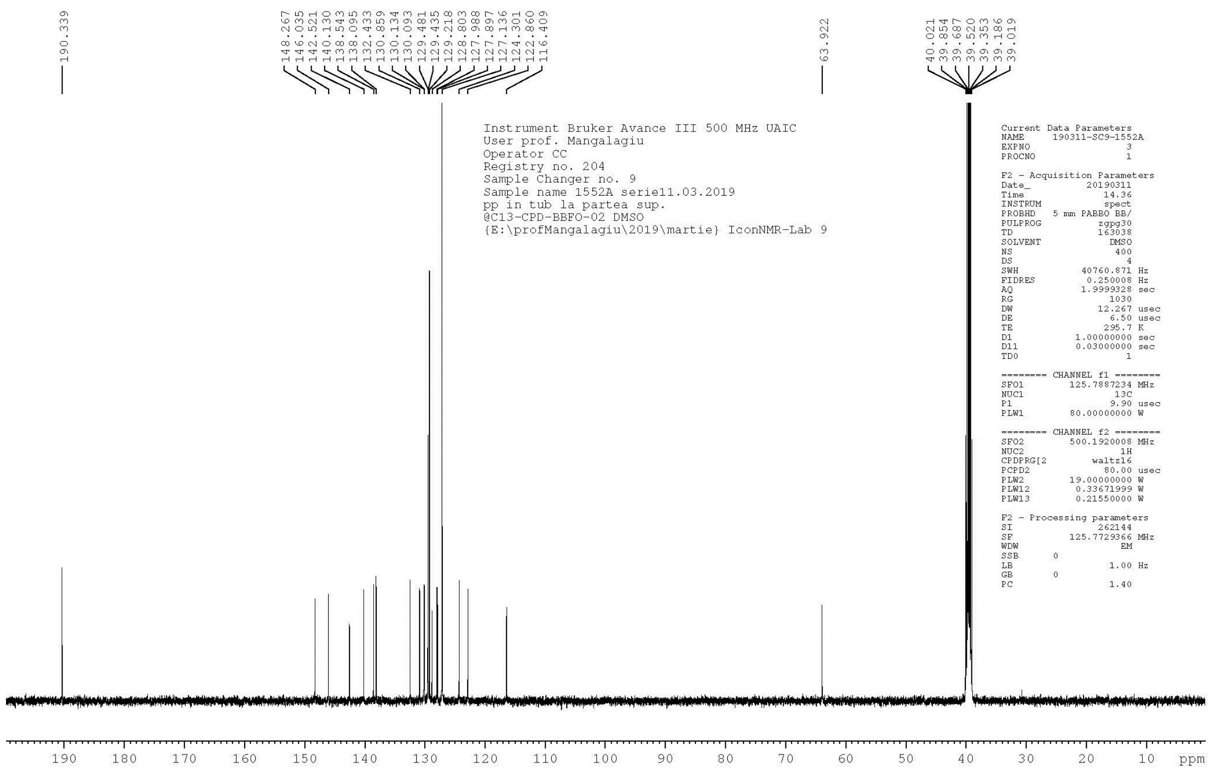

Figure S24. ^{1}H-NMR spectrum of 1-(4-cyanophenacyl)benzo[f]quinolin-1-ium bromide (**3k**)

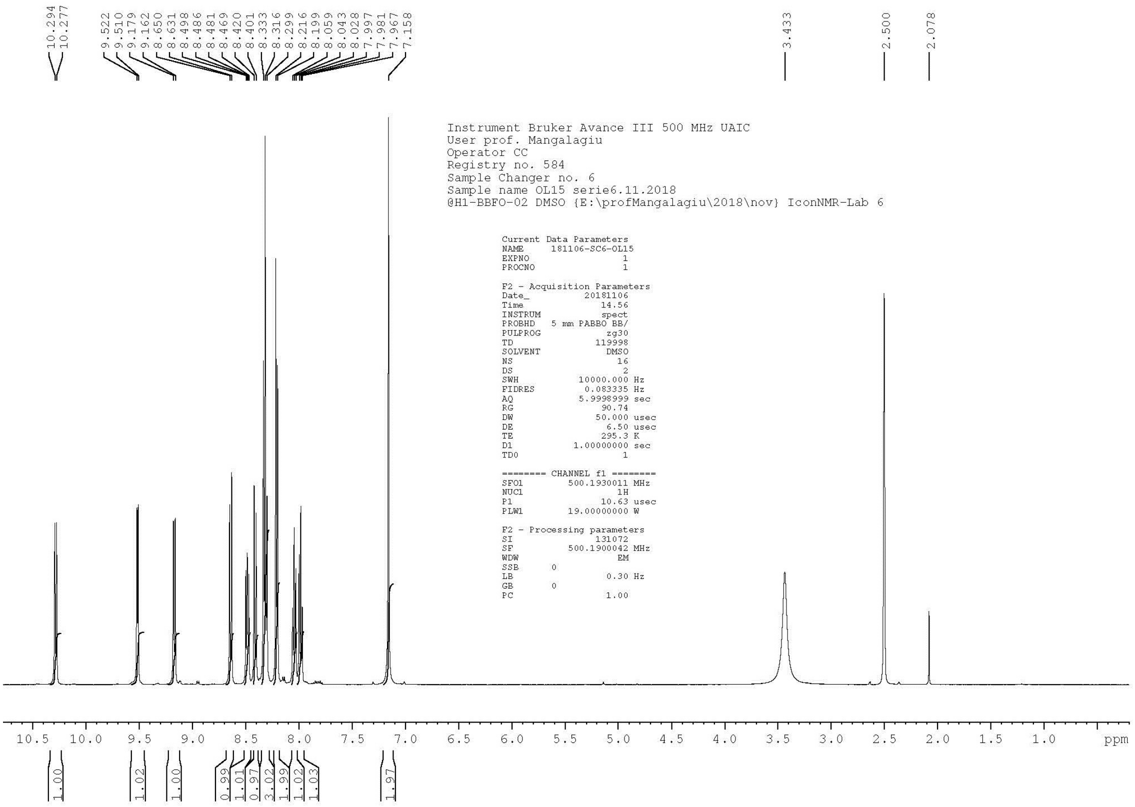

Figure S25. ^{13}C-NMR spectrum of 1-(4-cyanophenacyl)benzo[f]quinolin-1-ium bromide (**3k**)

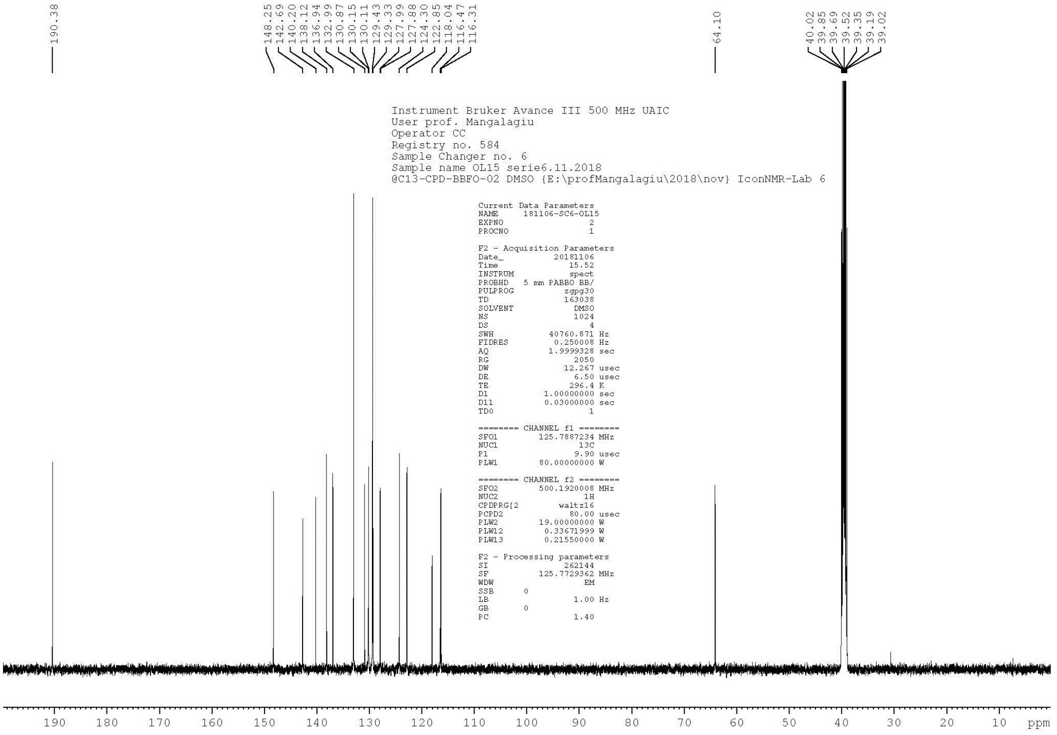

Figure S26. ^{1}H-NMR spectrum of 1-(4-nitrophenacyl)benzo[f]quinolin-1-ium bromide (**3l**)

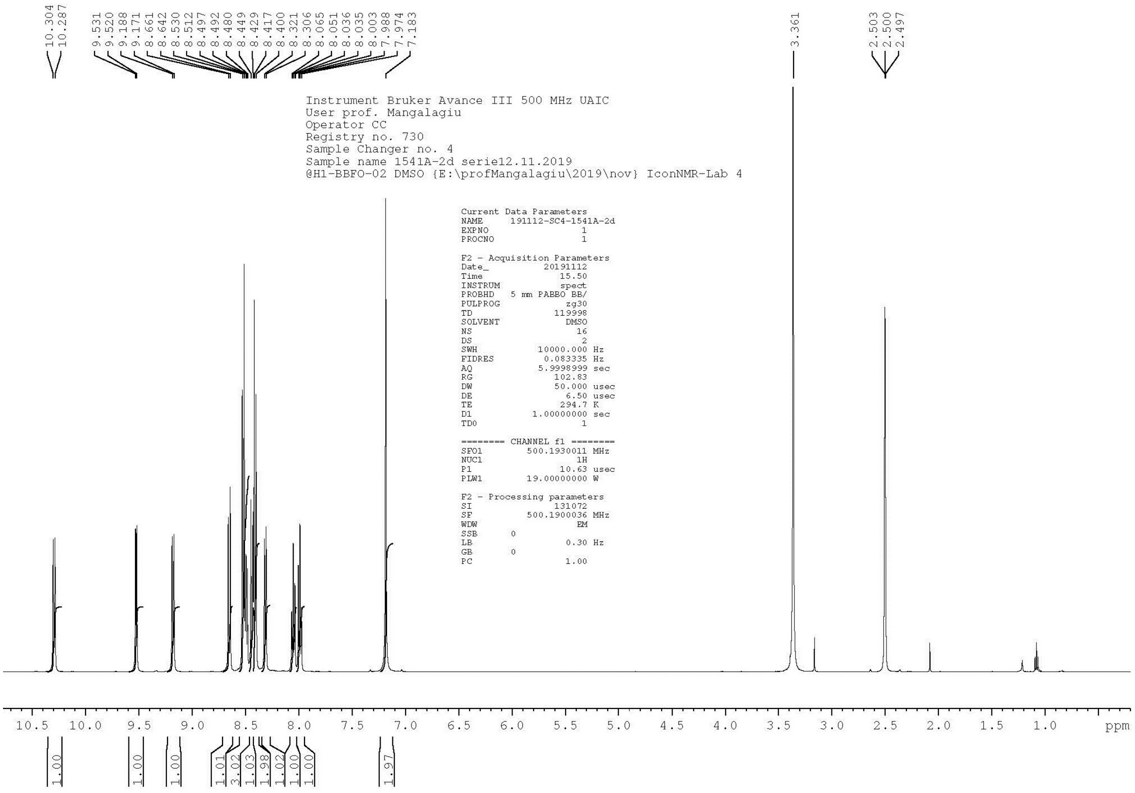

Figure S27. ^{13}C-NMR spectrum of 1-(4-nitrophenacyl)benzo[f]quinolin-1-ium bromide (**3l**)

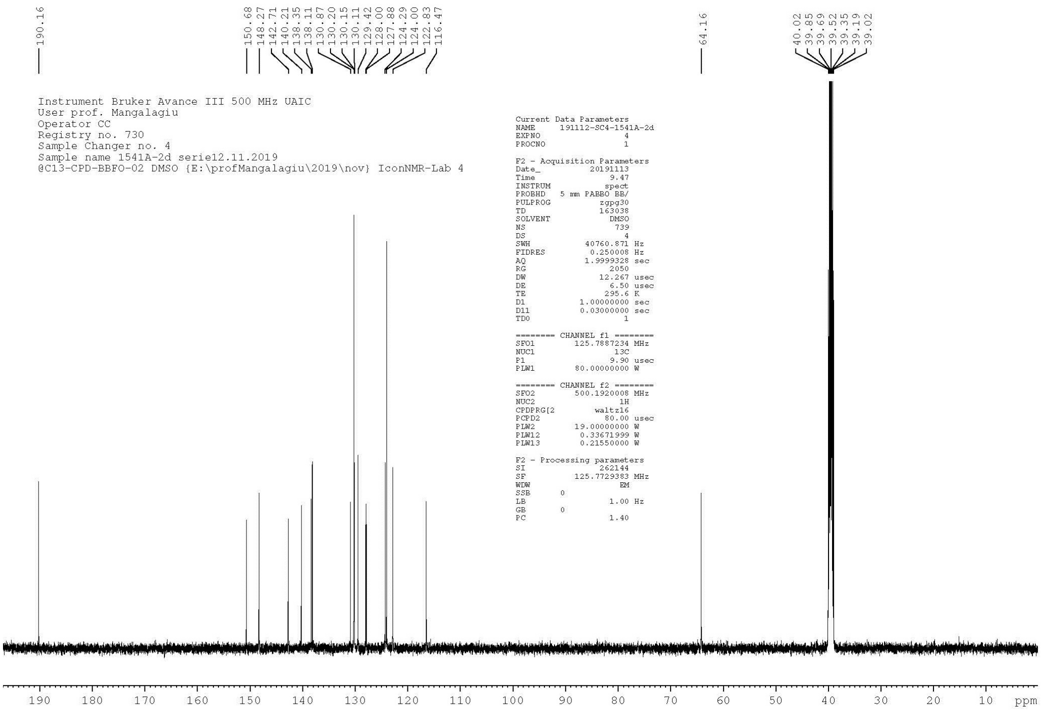

Figure S28. ^{1}H-NMR spectrum of 1-(4-bromophenacyl)benzo[f]quinolin-1-ium bromide (**3m**)

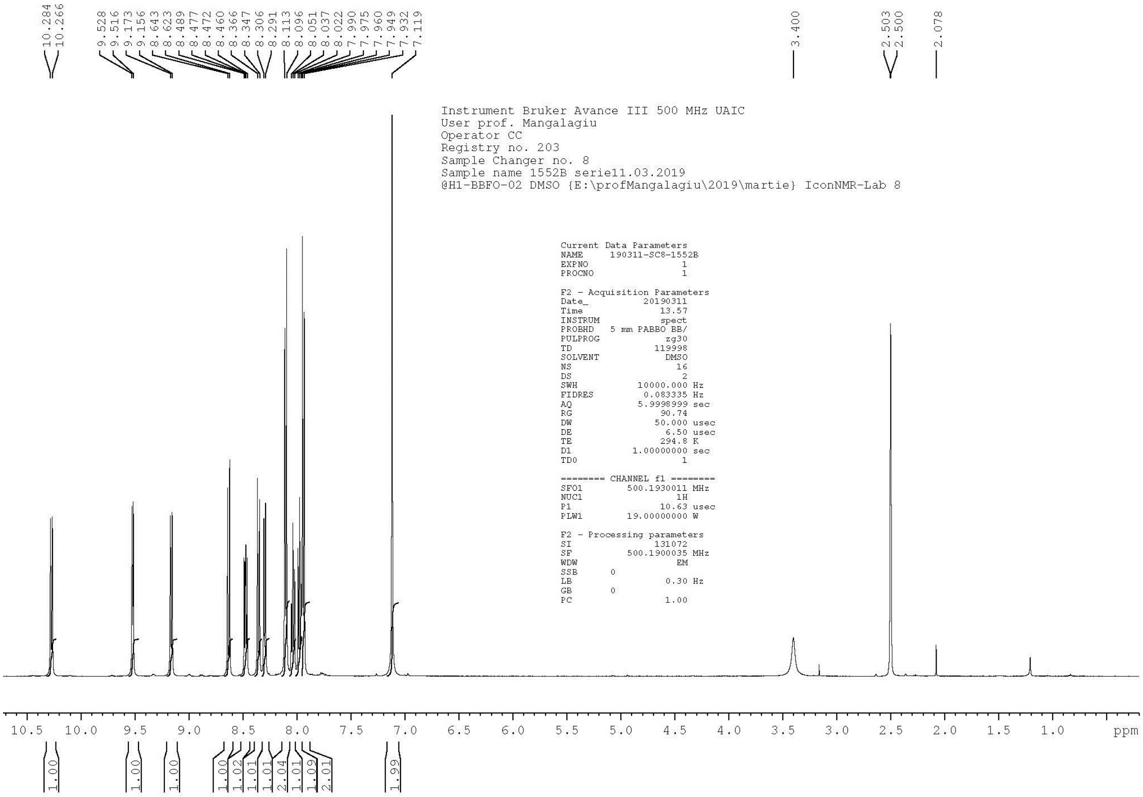

Figure S29. ^{13}C-NMR spectrum of 1-(4-bromophenacyl)benzo[f]quinolin-1-ium bromide (**3m**)

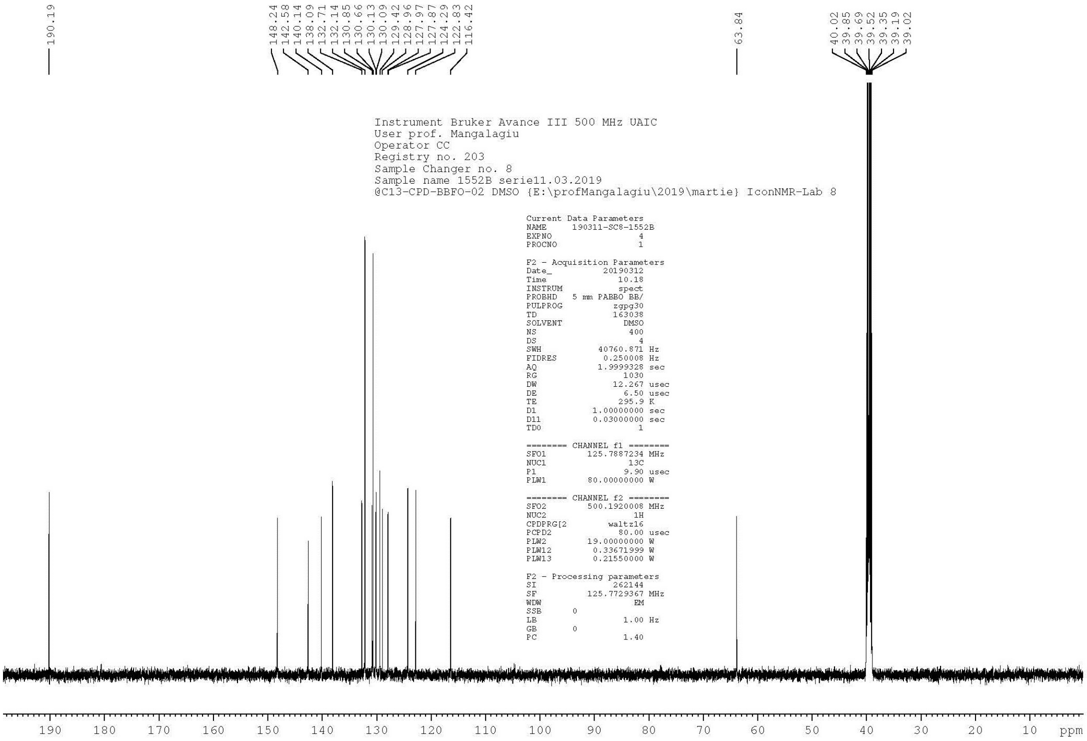

Figure S30. ^{1}H-NMR spectrum of 1-(4-chlorophenacyl)benzo[f]quinolin-1-ium bromide (**3n**)

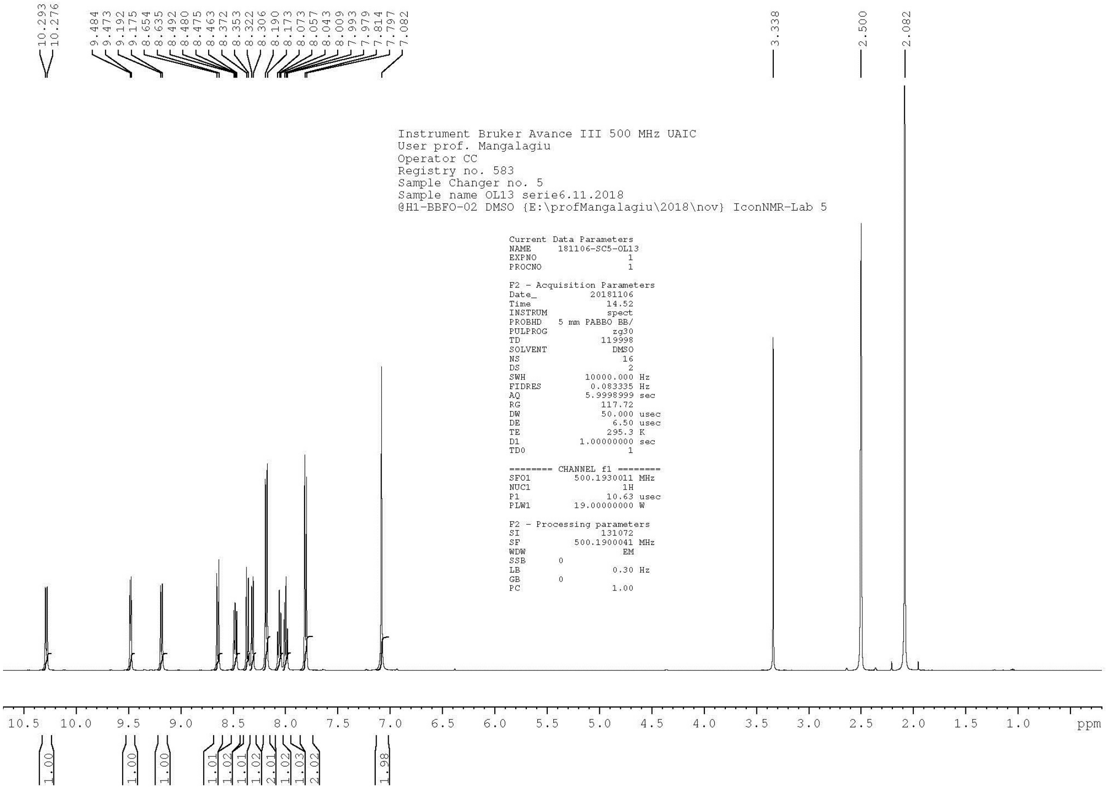

Figure S31. ^{13}C-NMR spectrum of 1-(4-chlorophenacyl)benzo[f]quinolin-1-ium bromide (**3n**)

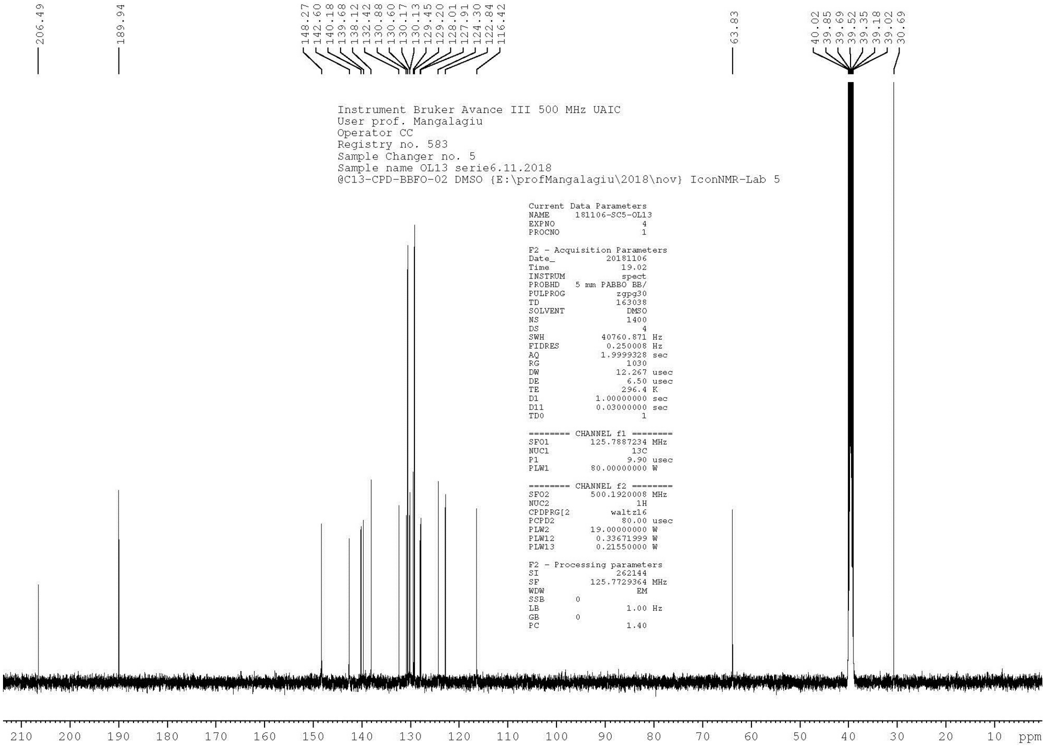

Figure S32. ^{1}H-NMR spectrum of 1-(4-fluorophenacyl)benzo[f]quinolin-1-ium bromide (**3o**)

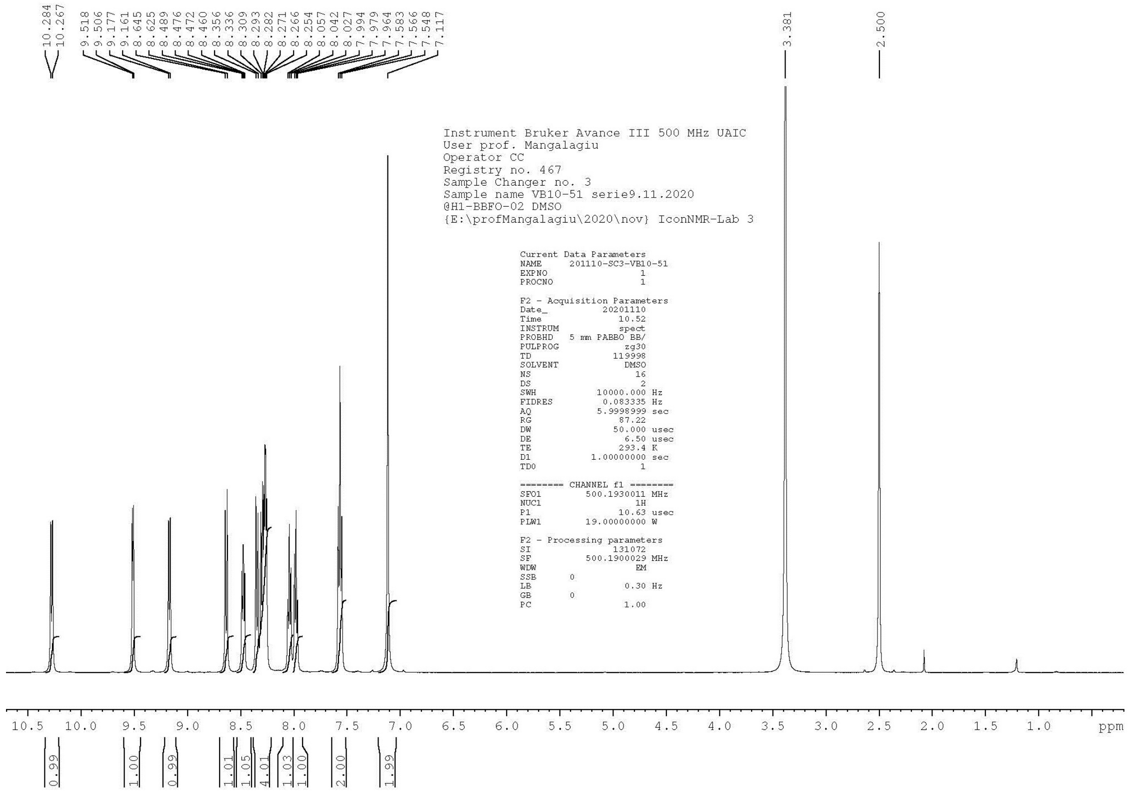

Figure S33. ^{13}C-NMR spectrum of 1-(4-fluorophenacyl)benzo[f]quinolin-1-ium bromide (**3o**)

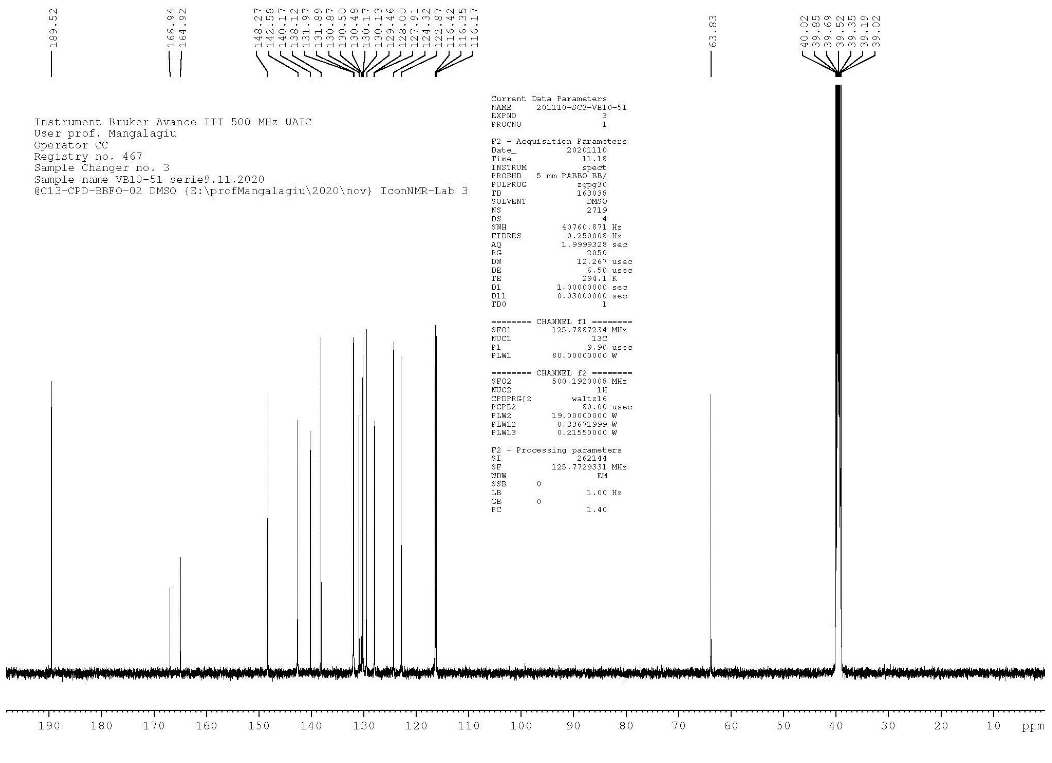

Supplement: Supplementary file 1 [file pharmaceuticals-14-00335-s001.pdf]
